# Transcriptional Insight Into *Brassica napus* Resistance Genes *LepR3* and *Rlm2*-Mediated Defense Response Against the *Leptosphaeria maculans* Infection

**DOI:** 10.3389/fpls.2019.00823

**Published:** 2019-07-02

**Authors:** Tengsheng Zhou, Wen Xu, Arvind H. Hirani, Zheng Liu, Pham Anh Tuan, Belay T. Ayele, Fouad Daayf, Peter B. E. McVetty, Robert W. Duncan, Genyi Li

**Affiliations:** ^1^Department of Plant Science, University of Manitoba, Winnipeg, MB, Canada; ^2^Crop Designing Centre, Henan Academy of Agricultural Sciences, Zhengzhou, China

**Keywords:** *Brassica napus*, *Leptosphaeria maculans*, blackleg disease, defense response, RNA-sequencing, *LepR3* and *Rlm2*, gene expression network

## Abstract

The phytopathogenic fungus *Leptosphaeria maculans* causes the blackleg disease on *Brassica napus*, resulting in severe loss of rapeseed production. Breeding of resistant cultivars containing race-specific resistance genes is provably effective to combat this disease. While two allelic resistance genes *LepR3* and *Rlm2* recognizing *L. maculans* avirulence genes *AvrLm1* and *AvrLm2* at plant apoplastic space have been cloned in *B. napus*, the downstream gene expression network underlying the resistance remains elusive. In this study, transgenic lines expressing *LepR3* and *Rlm2* were created in the susceptible “Westar” cultivar and inoculated with *L. maculans* isolates containing different sets of *AvrLm1* and *AvrLm2* for comparative transcriptomic analysis. Through grouping the RNA-seq data based on different levels of defense response, we find *LepR3* and *Rlm2* orchestrate a hierarchically regulated gene expression network, consisting of induced ABA acting independently of the disease reaction, activation of signal transduction pathways with gradually increasing intensity from compatible to incompatible interaction, and specifically induced enzymatic and chemical actions contributing to hypersensitive response with recognition of *AvrLm1* and *AvrLm2*. This study provides an unconventional investigation into *LepR3* and *Rlm2*-mediated plant defense machinery and adds novel insight into the interaction between surface-localized receptor-like proteins (RLPs) and apoplastic fungal pathogens.

## Introduction

Rapeseed (*Brassica napus* L.) is one of the main sources for plant-produced oil, which provides healthy edible oil for human and feedstock for biodiesel fuel (Ecke et al., 1995; Bouaid et al., 2009). *Leptosphaeria maculans* (anamorph *Phoma lingam*), the causal agent of blackleg disease on *B. napus*, poses a continual threat to canola/rapeseed production (West et al., 2001). Like other dothideomycete phytopathogenic fungi, *L. maculans* enters the host via natural openings such as stomata and wounds (Howlett et al., 2001). However, *L. maculans* has a complex life cycle which begins with a brief invasion of cotyledons, followed by a long endophytic and asymptomatic period in leaves and stem tissue, leading to necrotrophic growth and finally causing the crown canker and yield loss (Howlett et al., 2001). The primary management measure to prevent crop loss in rapeseed is to integrate multiple strategies consisting of crop rotation, fungicide use and introgression of qualitative or quantitative resistance (West et al., 2001; Rimmer, 2006).

Several major loci responsible for race-specific resistance to *L. maculans* have been mapped in *B. napus*, with most loci clustering on chromosomes A7 and A10 of A genome (Delourme et al., 2006). Until now, only two allelic resistance genes *LepR3* (or *BLMR1.1* with Accession No. JQ979409) and *Rlm2* on the chromosome A10 have been cloned from two cultivars (Long et al., 2011; Larkan et al., 2013, 2015). The hemibiotrophic lifestyle is exhibited by *L. maculans* during the initial foliar infection. The successful infection is determined by the hemibiotrophic transition, during which an arsenal of small secretion proteins (SSPs) are deployed to manipulate the host immune response toward the necrotrophic growth (Haddadi et al., 2016). In *L. maculans*, eight avirulence (*Avr*) genes (*AvrLm1, AvrLm2, AvrLm3, AvrLm4-7, AvrLm5, AvrLm6*, and *AvrLm1*) that provoke the *B. napus* race-specific resistance have been cloned and all of these *Avr* genes encode SSPs (Gout et al., 2006; Fudal et al., 2007; Parlange et al., 2009; Balesdent et al., 2013; Van de Wouw et al., 2014; Ghanbarnia et al., 2015; Plissonneau et al., 2016). Among cloned *Avr* genes, *AvrLm1* and *AvrLm2* have been demonstrated to trigger resistance by *LepR3* and *Rlm2* in *B. napus*, respectively (Long et al., 2011; Larkan et al., 2013, 2014), with lack of details describing the interaction machinery.

To date, knowledge regarding the plant resistance machinery against pathogen invasion is mostly gained based on studies performed on several model plant pathosystems including *Arabidopsis*, tomato, potato and rice (Jones et al., 2013), leading to the current and widely accepted paradigm for describing plant immunity, the zigzag model (Jones and Dangl, 2006; Cook et al., 2015). This model is defined by a clear dichotomy of two-layered defense responses, subsequently activated via the extracellular detection of pathogen-associated molecular patterns (PAMPs) by surface-localized receptor-like proteins (RLPs) and intracellular recognition of pathogen effector proteins by nucleotide-binding-leucine-rich repeat (NB-LRRs) receptors, which are, respectively, termed as pattern-triggered immunity (PTI) and effector-triggered immunity (ETI). However, due to the limited diversity of research model organisms, the zigzag model is insufficient to cover the diversity of pathogen-host interplay mechanism due to species-specificity (Hacquard et al., 2017). As an apoplastic fungal pathogen, *L. maculans* only enters the plant cells after successfully cracking down the host immune response via SSPs at the apoplastic space. Thus, the effective *B. napus* resistance is activated upon recognition of pathogen SSPs by surface-localized RLPs, as exampled by LepR3 and Rlm2 that further transmit the defense signals via engaging the other surface-localized receptor-like kinases (RLKs) (Larkan et al., 2015; Ma and Borhan, 2015). This is analogous to PAMP recognition receptors (PRRs) as members of RLPs that capture chemical cues associated with fungal structures to trigger the basal resistance (Stotz et al., 2014). Indeed, either PAMP or SSP-interacting RLPs form complex with the receptor-like kinase SOBIR1 for structural stabilization and signal trafficking at the membrane areas (Stotz et al., 2014). In contrast to the typically qualitative nature of intracellular *R* gene-mediated ETI to cause the hypersensitive reaction to restrain the pathogen infection, the host response upon the apoplastic recognition specificity in the *B. napus*–*L. maculans* pathosystem engages a more quantitative manner that still allows fungal entry into the host tissues to propagate (Huang et al., 2006).

Both host recognition of PAMPs and SSPs cause substantial transcriptional reprogramming that shares similar signal pathways but differs at the expressional magnitudes (Dodds and Rathjen, 2010). Given the genomic complexity and underdeveloped genetic manipulation techniques for *B. napus*, RNA-sequencing (RNA-seq)-based comparative transcriptomic analysis has been extensively used to study the gene expression network governing the defense response against *L. maculans* (Lowe et al., 2014; Haddadi et al., 2016; Becker et al., 2017). Recent studies have revealed several prominent groups of genes transcriptionally induced, likely acting in a subsequent order to orchestrate cellular responses, during the resistance reaction to *L. maculans* (Becker et al., 2017). These genes are involved in extracellular signal detection via surface RLPs, intracellular signal trafficking, plant hormone synthesis, defense response-related transcriptional activities, cell wall enforcement enzymes, and sulfate-related secondary metabolism. However, these genes were identified via the transcriptomic comparison between susceptible and resistant cultivars that differ in genetic backgrounds and may cause bias on downstream analysis. In addition, the data interpretation based on the gene-for-gene doctrine tends to marginalize the effect of surface-localized RLPs as receptors to detect fungal invasion, during the compatible interaction. Studies have revealed that the distinction between the host susceptible and resistant responses to pathogen infection is largely due to the quantitative difference on output signal intensity of defense-related transcriptome (Tao et al., 2003; Tsuda and Katagiri, 2010). Here, we assume that *LepR3* and *Rlm2* are involved in evoking the basal defense in the PTI network during the compatible interaction, while the signal intensity can pass the threshold resulting in the resistant response following the recognition of *AvrLm1* and *AvLm2* during the incompatible interaction.

Global transcriptome profiling on *LepR3* and *Rlm2* transgenic lines challenged by *L. maculans* isolates with different *AvrLm1* and *AvrLm2* patterns revealed the detailed machinery of *LepR3* and *Rlm2* in regulating plant defense during the compatible and incompatible interaction. Genes contributing to *LepR3* and *Rlm2*-mediated resistance were identified and constitute the gene expression network based on expression patterns and functional classification. Lastly, the RNA-seq-deduced *LepR3* and *Rlm2* regulatory machinery was phenotypically tested by the infection site measurement of hormone levels (abscisic acid and salicylic acid) and hydrogen peroxide production.

## Materials and Methods

### Plant and Fungal Materials

The susceptible *B. napus* cultivar “Westar” and four *L. maculans* isolates 03-42-06 (*AvrLm1* and *avrLm2*), 87-41 (*AvrLm1* and *AvrLm2*), 03-15-03 (*avrLm1* and *AvrLm2*), and PG4-1-M (*avrLm1* and *avrLm2*) were used in this study (Supplementary Table S1). For four *L. maculans* isolates, 87-41 was firstly collected from blackleg infected *Brassica oleracea* (Yu et al., 2008) and the other three isolates 03-42-06, 03-15-03 and PG4-1-M were recently collected from fields in western Canada. To determine the genotypes of six cloned avirulent genes (*AvrLm1, AvrLm2, AvrLm4-7, AvrLm6, AvrLm1*, and *AvrLm5*) in *L. maculans* isolates, genomic DNA was extracted from mycelia collected on 5-day-old PDA plates using a Qiagen DNeasy Plant Mini Kit (Qiagen, Canada) and subjected to PCR and sequencing analysis using primers in Supplementary Table S5. Seedlings of *B. napus* were grown in 96-cell trays in a monitored growth chamber (21°C for 16 h light and 18°C for 8 h dark cycles) for 1 week using sunshine mix four soil (Planet Natural, Canada). To acquire *L. maculans* pycnidiospores for inoculation, 100 μl 10^8^ spores/mL suspension was evenly spread on the V8 juice agar plates and exposed to constant light for 1 week to induce sporulation at room temperature. True leaves were removed from plants to avoid cotyledon senescence after inoculation. For *L. maculans* inoculation, wounded sites made by a needle were made on each side of the primary vein in each cotyledon of 7-day-old seedlings and 10 μl of 2 × 10^7^ spores/mL suspension was applied to each wounding site. For tissue collected for RNA-seq, four inoculations were made on each side of the primary vein in each cotyledon. To better observe hydrogen peroxide accumulation, infiltration inoculation was applied according to the description in (Šašek et al., 2012) on 7-day-old *B. napus* seedlings. The inoculated seedlings were kept in the same moisture chamber used for plant growth, and disease development was monitored accordingly.

### Vector Construction and Creation of Transgenic Lines

The *LepR3* (or *BLMR1.1* accession No. JQ979409) in “Surpass 400” and the homologous gene *blmr1* (accession No. JQ979415.1) in “Westar” were cloned in this lab. To make the expression vectors under the native promoters, the primer set Le3_Rl2_F and Le3_Rl2_R (Supplementary Table S5) were used to amplify the fragments including the promoter and open reading frame (ORF) from the two resistant cultivars “Surpass 400” (*LepR3*) and “Glacier” (*Rlm2*), respectively, and inserted into the binary vector pCAMBIA1300^1^ to produce two complementary constructs pCAMBIA1300-LepR3 and pCAMBIA1300-Rlm2. To make the overexpression constructs for *LepR3*, total RNA from “Surpass 400” seedlings was extracted using TRIzol^®^ Reagent (Thermo Fisher Scientific, United States) following the manufacturer’s instructions. Full-length cDNA was synthesized using SuperScript II reverse transcriptase kit (Thermo Fisher Scientific, United States) and amplified using the primer set 35S_Le3_F and 35S_Le3_R (Supplementary Table S5). The full-length cDNA of *LepR3* was cloned into the binary vector pBI121 to make the overexpression construct pBI121-LepR3, whose expression is driven by the 35S promoter. The three constructs were used to perform plant transformation into “Westar” as described previously (Liu et al., 2011). Seeds from 40 transgenic T0 generation plants for TLepR3 transformation, 20 for TRlm2 and 10 for T35SLepR3 were produced. Single insertion transgenic lines were identified through analysis of segregation of 48 T1 progeny individuals for each T0 line. Homozygous lines were produced from single insertion transgenic lines through selfing and used in the gene expression analysis.

### RNA Extraction, cDNA Library Construction, RNA-Sequencing, and RT-qPCR

Five independent inoculated *B. napus* seedlings for each biological replicate were collected and combined for RNA extraction. Total RNA was extracted using TRIzol^®^ Reagent (Thermo Fisher Scientific, United States) following the manufacturer’s instructions and treated with the DNA-free DNA Removal Kit (Thermo Fisher Scientific, United States) to remove genomic DNA contamination. In total, 60 samples (including mock and pathogen-inoculated *B. napus* samples) were checked for RNA integrity number (>6.5) by an Agilent Bioanalyzer 2100 and subjected to library construction using the TruSeq RNA Library Preparation Kit v2 (Illumina, United States) following the manufacturer’s instructions. All samples were sequenced on an Illumina HiSequation 2500 platform at the Genome Québec Innovation Centre, McGill University. The sequencing was performed as paired-end reads that were 2 bp × 100 bp in length. All raw RNA-Seq read data are deposited in the NCBI Short Read Archive^2^ under the BioProject accession number PRJNA378851.

Isolated total RNA was used to synthesize cDNA library using the SuperScript^TM^ IV First-Strand Synthesis System (Thermo Fisher Scientific). SYBR^®^ Green Master Mix (Bio-Rad) was used to conduct RT-qPCR in a CFX96 Thermal Cycler (Bio-Rad) according to manufacturer’s instruction. The gene specific primers for RT-qPCR analysis were listed in Supplementary Table S5. The actin gene (Gene ID BnaC02g00690D) was used as the internal control for normalization. The 2^-ΔΔCT^ method was used to analyze the data (Livak and Schmittgen, 2001).

### RNA-Seq Data Mapping, Gene Expression Analysis, and Functional Annotation

High-quality reads of each sample were filtered out using the NGS QC tool kit (Patel and Jain, 2012) according to default parameters. Bowtie2 (Langmead and Salzberg, 2012) was used to map RNA-seq reads separately to the *B. napus* (Wang et al., 2011) and *L. maculans* (Rouxel et al., 2011) genomes following the instructions, and read counting of each transcript was carried out with HTSeq-count (Anders et al., 2015). The DESeq2 package (Love et al., 2014) was used to normalize the raw reads mapping to the *B. napus* reference genome and identify differentially expressed genes (DEGs) in different comparisons. Genes with a reported padj value <0.05 were regarded showing a statistically significant difference between samples. The *B. napus* DEGs were annotated by searching the open reading frame (ORFs) sequences against the nr protein database using an E-value cut-off of 10^-5^ and reporting the maximum “hit” sequence per query.

### Functional Classification Based on Gene Ontology and MapMan Enrichment and Heatmap Generation

GOstats package installed under the R/Bioconductor environment was used to enrich GO terms for identified DEGs in each gene cluster (Falcon and Gentleman, 2006), as the corresponding *P*-value was set (*P* < 0.05). To reduce the redundant GO terms in the same DEGs list, REViGO analysis^3^ was used to slim the enriched GO terms based on the medium-sized similarity. The MapMan tool (Thimm et al., 2004) was used to facilitate the assignment of identified *B. napus* DEGs into functional categories (bins). A MapMan mapping file that mapped the *B. napus* genes into bins via hierarchical ontologies through the searching of a variety of reference databases was generated using the Mercator tool^4^. The identified DEGs were matched to the mapping file by the MapMan software and assigned into ontology bins according to default parameters. To generate heatmaps showing the expression changes of identified DEGs, the log2-fold change values for each gene was calculated by making the sample comparison against the water control “Westar” and transformed into the heatmap using the pheatmap R package (pheatmap version, 1.0.8). Similarities in the shape of expression profiles with regard to the up- and down-regulated DEGs were measured by the correlation index *R* as defined in (Tao et al., 2003) 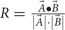 where $\vec{A}$ and $\vec{B}$ were a pair of sample data sets in the linear space with dimensions equal to the number of DEGs. The average amplitude of each up- or down-regulated expression profile was presented as the average number of the log2-transformed fold change values for each data set.

### Hydrogen Peroxide Detection, Abscisic Acid, and Salicylic Acid Content Determination

For detecting hydrogen peroxide, the staining solution was prepared by dissolving 10 mg diaminobenzidine tetrahydrochloride hydrate (DAB) (Sigma, United States) in 15 mL 10 mM pH 7.5 Tris buffer in a 50 mL falcon tube. Detached cotyledons after 2- and 4-day infiltrated-inoculation were immersed in the staining solution and infiltrated under vacuum in the 50 mL falcon tube in darkness at room temperature until reddish-brown staining was detected. Chlorophyll was removed in fixation solution. Photos were taken after cotyledons were rehydrated in gradient ethanol solutions. The intensity of oxidized DAB in detached cotyledons was quantified by using the ImageJ software after collecting 10 samples with three independent replications (Schneider et al., 2012). The mean pixel intensity in staining regions was measured, and the reduction of pixel intensity was proportional to the increase of oxidized brown DAB precipitation.

The levels of abscisic acid and salicylic acid were determined from freeze-dried TRlm2, T35SLepR3 and “Westar” cotyledons infected with 03-42-06 isolate and water as the control treatment. Five cotyledons were collected for each sample in an individual replication with three independent replications. The lyophilized tissues were homogenized with 80% (v/v) acetonitrile containing 1% (v/v) acetic acid and the internal standards, and all the subsequent procedures for the extraction and purification of ABA and SA was carried out as described previously (Kanno et al., 2010; Son et al., 2016). Measurement of the levels of ABA and SA was performed with liquid chromatography-tandem mass spectrometry system (Agilent 1260-6430) using conditions described previously (Yoshimoto et al., 2009).

## Results

### Interaction Analysis of *LepR3* and *Rlm2* Transgenic Lines With *L. maculans* Isolates Containing Different *AvrLm1* and *AvrLm2* Patterns

**FIGURE 1:**
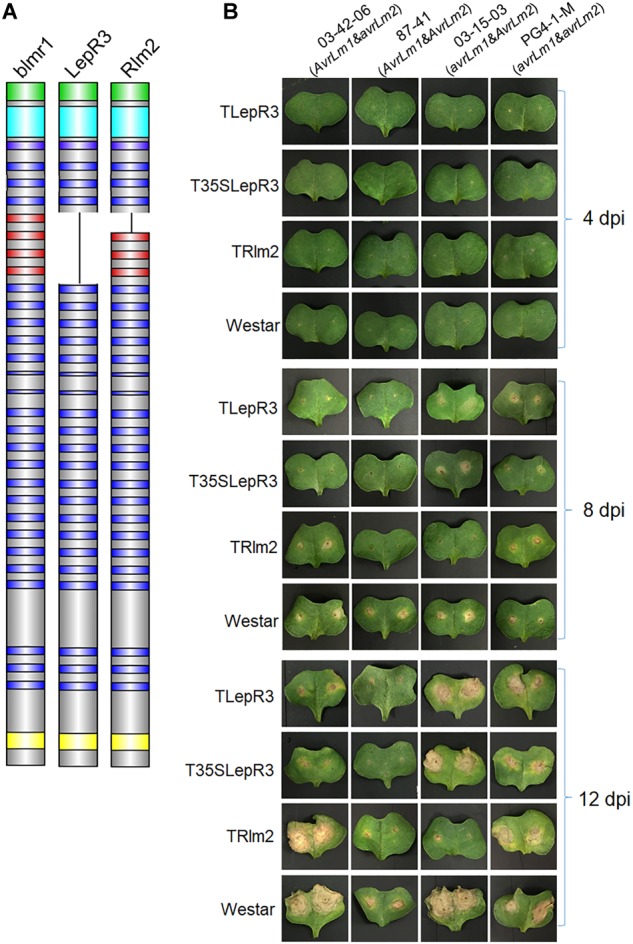
Phenotypic interaction of TLepR3, T35SLepR3, and TRlm2 with *L. maculans* isolates containing different *AvrLm1* and *AvrLm2* patterns. **(A)** Schematic diagrams representing the blmr1 protein in *B. napus* “Westar” cultivar and its homologs LepR3 and Rlm2 in “Surpass 400” and “Glacier” cultivars. The domains are colored as the N-terminus secretion peptide (green), the leucine-rich repeat N-terminal domain (cyan), the leucine-rich repeats (LRRs) (blue and red), and the C-terminus transmembrane domain (TM) (yellow). The missing LRRs in LepR3 are colored in red. **(B)** Cotyledons of 7-day-old seedlings of transgenic lines (TLepR3, T35SLepR3, and TRlm2) and “Westar” were inoculated with *L. maculans* isolates 03-42-06 (*AvrLm1* and *avrLm2*), 87-41 (*AvrLm1* and *AvrLm2*), 03-15-03 (*avrLm1* and *AvrLm2*), and PG4-1-M (*avrLm1* and *avrLm2*). Photos were taken at 4, 8, and 12 dpi.

*LepR3* (or *BLMR1.1* with Accession No. JQ979409) and *Rlm2* as two allelic resistance genes in *B. napus* “Surpass 400” and “Glacier” cultivars cause *B. napus* resistance against *L. maculans* containing *AvrLm1* and *AvrLm2*, respectively (Long et al., 2011; Larkan et al., 2013, 2014, 2015). *blmr1* (accession No. JQ979415.1) is the homologous gene of *LepR3* and *Rlm2* in the “Westar” cultivar that is susceptible to *L. maculans* isolates. The amino acid sequence analysis and alignment of three homologs showed that they are distinct in the N-terminal leucine-rich repeat motifs (Figure 1A and Supplementary Figure S1). Gene expression constructs for *LepR3* and *Rlm2* driven by the native promoters were created and transformed into “Westar” and produced two transgenic lines namely TLepR3 and TRlm2 (Figure 1B). To test whether the enhanced expressional level of resistance gene can affect the plant defense response, the overexpression vector for *LepR3* driven by the 35S promoter was also created and transformed into “Westar” to produce the transgenic line with higher expression of *LepR3*, namely T35SLepR3.

To differentiate the defense response between compatible and incompatible interaction, four isolates with different patterns of *AvrLm1* and *AvrLm2* were used for inoculation on all *B. napus* lines (Figure 1B and Supplementary Table S1). A chlorotic ring around the inoculation site formed and became visible after 4-day-post inoculation (dpi) on all inoculated lines. The extension of infected areas on “Westar” cotyledons led to the formation of necrotic lesions at 8 dpi and enlarged at 12 dpi. Inoculation with isolates 87-41 and PG4-1-M showed less severe symptoms on “Westar,” suggesting the weak virulence. Other compatible interaction included the inoculation of PG4-1-M on TLepR3, T35SLepR3, and TRlm2, the inoculation of 03-15-03 on TLepR3 and T35SLepR3 and the inoculation of 03-42-06 on TRlm2. Lesion expansion on TLepR3 and T35SLepR3 inoculated with isolate 03-42-06 or 87-41 was contained for the incompatible interaction at 8 and 12 dpi. The restriction of disease progress was similar for the incompatible interaction on TRlm2 inoculated with 87-41 or 03-15-03 at 8 and 12 dpi.

### RNA-Seq Analysis of Transcriptomic Dynamics in the *B. napus*–*L. maculans* Pathosystem During the Early Compatible and Incompatible Interaction

To dissect the defense response mediated by *LepR3* and *Rlm2* during early compatible and incompatible interaction, RNA-seq was employed to analyze the transcriptome of *B. napus* transgenic lines challenged by four *L. maculans* isolates at 4 dpi (Table 1). Three biological replicates were conducted for all inoculation. In total, 60 RNA samples were subjected to dual RNA-seq, which generated 485 million reads mapped to the *B. napus* genome (Supplementary Table S2). Because of a large number of samples in this study, biological replication among samples collected from the same *B. napus* line inoculation was determined by principal component analysis (PCA) (Supplementary Figure S2), which displayed a clear cluster of replicates inoculated with the same isolate.

**Table 1:** Experiments for RNA-seq performed in this study.

		Time
*L. maculans*	*B. napus*	point	Number of	Disease
isolates^a^	lines^b^	(dpi)	replicates	development
03-42-06				
(*AvrLm1* and *avrLm2*)	TLepR3	4	3	R
	T35SLepR3	4	3	R
	TRlm2	4	3	S
	Westar	4	3	S
87-41				
(*AvrLm1* and *AvrLm2*)	TLepR3	4	3	R
	T35SLepR3	4	3	R
	TRlm2	4	3	R
	Westar	4	3	S
03-15-03				
(*avrLm1* and *AvrLm2*)	TLepR3	4	3	S
	T35SLepR3	4	3	S
	TRlm2	4	3	R
	Westar	4	3	S
PG4-1-M				
(*avrLm1* and *avrLm2*)	TLepR3	4	3	S
	T35SLepR3	4	3	S
	TRlm2	4	3	S
	Westar	4	3	S
Water				
	TLepR3	4	3	
	T35SLepR3	4	3	
	TRlm2	4	3	
	Westar	4	3	

*^*a*^*Leptosphaeria maculans* isolates with four *AvrLm1* and *AvrLm2* genotypes were used to inoculate at 2 × 10^7^ spores/ml, which were collected from 7-day-old V8 plates under constant light illumination. ^*b*^All *B. napus* lines are created in the “Westar” background. R, resistance; S, susceptible.*

### Dissection of *LepR3* and *Rlm2*-Mediated Resistance Response via Grouping the Inoculation of Isolates 03-42-06 (*AvrLm1* and *avrLm2*) and 03-15-03 (*avrLm1* and *AvrLm2*) on “Westar,” T35SLepR3, and TRlm2

**FIGURE 2:**
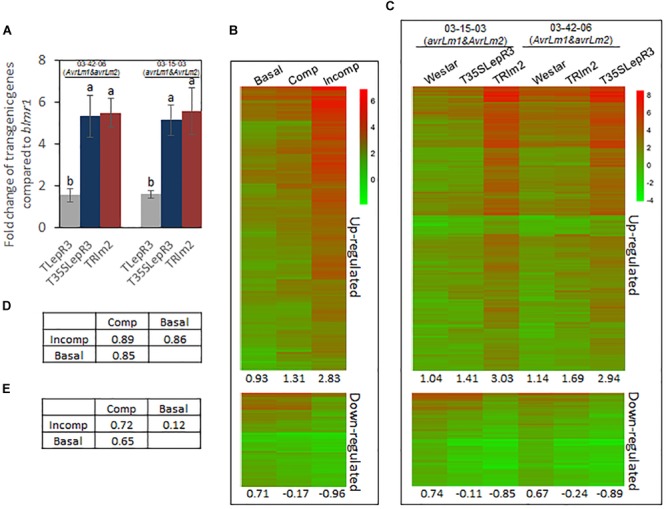
Induction of defense transcriptome by *LepR3* and *Rlm2* during the compatible and incompatible interaction. **(A)** Validation of *LepR3* or *Rlm2* expression level compared to the endogenous *blmr1* in transgenic lines TLepR3, T35SLepR3, and TRlm2 in response to the infection of *L. maculans* isolate 03-42-06 (*AvrLm1* and *avrLm2*) and 03-15-03 (*avrLm1* and *AvrLm2*) at 4 dpi using RT-qPCR. Error bars mean standard deviation based on data collected on three replicates in three independent experiments. Different letters indicate significant differences from each other, α = 0.05 (ANOVA). **(B,C)** Hierarchically clustering heatmaps showing the expression profiles of identified 1,016 up-regulated and 332 down-regulated genes for *LepR3* and *Rlm2* resistance. RNA-seq data collected on *L. maculans* isolates (03-42-06 and 03-15-03)-inoculated T35SLepR3, TRlm2, and “Westar” were divided into three groups, including inoculation on “Westar” by 03-42-06 (*AvrLm1* and *avrLm2*) and 03-15-03 (*avrLm1* and *AvrLm2*) as “Basal” group, inoculation on T35SLepR3 by 03-15-03 (*avrLm1* and *AvrLm2*) and TRlm2 by 03-42-06 (*AvrLm1* and *avrLm2*) showing compatible interaction as “Comp” group or inoculation on T35SLepR3 by 03-42-06 (*AvrLm1* and *avrLm2*) and TRlm2 by 03-15-03 (*avrLm1* and *AvrLm2*) showing incompatible interaction as “Incomp” group. DEGs were expressed in “Basal,” “Comp,” and “Incomp” **(B)**, and in “Westar,” T35SLepR3, and TRlm2 inoculated by isolate 03-15-03 (*avrLm1* and *AvrLm2*) or 03-42-06 (*AvrLm1* and *avrLm2*) **(C)**. The heatmaps were generated based on log2-transformed fold change values comparing to the mock-inoculated “Westar.” The average profile amplitudes are indicated below the heatmaps. **(D,E)** Tables of pair-wise correlations for expression profiles of 1,016 up-regulated **(D)** and 332 down-regulated **(E)** genes in the “Basal,” “Comp,” and “Incomp.”

Due to the high sequence similarity between *LepR3, Rlm2*, and *blmr1* (Supplementary Figure S1), the expression level of each allele cannot be differentiated in the RNA-seq analysis. The expression level of *LepR3* or *Rlm2* in transgenic lines were determined using allele-specific primers in the RT-qPCR assay. Comparing to the endogenous *blmr1* of the background “Westar,” the expression level of *LepR3* in TLepR3 was about 1.6-time that of *blmr1* in response to 03-42-06 and 03-15-03 infection, while much higher expression level of the inserted transgene (*LepR3* or *Rlm2*) with around 5 to 6-time that of *blmr1* in TRlm2 and T35SLepR3 were detected (Figure 2A).

In this study, we intend to investigate the role of *LepR3* and *Rlm2* in both compatible and incompatible interaction. Due to equally aggressive virulence at 4 dpi between isolate 03-42-06 (*AvrLm1* and *avrLm2*) and 03-15-03 (*avrLm1* and *AvrLm2*) and similar expression level of transgenes between T35SLepR3 and TRlm2, inoculation on “Westar,” T35SLepR3, and TRlm2 by two isolates were classified into three groups according to presumed defense response levels (Supplementary Figure S3). The susceptible “Westar” response to *L. maculans* 03-42-06 and 03-15-03 was considered as the basal defense and data collected on inoculated “Westar” were assigned as “Basal” group. The second group included the compatible interaction on T35SLepR3 inoculated by 03-15-03 and TRlm2 inoculated by 03-42-06, and was named as “Comp.” The third group namely “Incomp” included the incompatible interaction on T35SLepR3 inoculated by 03-42-06 and TRlm2 inoculated by 03-15-03. Percentage of differentially expressed genes (DEGs) of pairwise comparison within each group was ∼0.3%, indicating low biological variation. The PCA analysis on RNA-seq data further revealed that six RNA-seq samples among each group are highly clustered (Supplementary Figure S3). Therefore, RNA-seq data collected within the same group were regarded as the biological replicates and pooled together for analysis.

### *LepR3* and *Rlm2* Modulate Increasing Scale of Defense Transcriptome From Compatible to Incompatible Interaction

To explore the regulatory machinery of *LepR3* and *Rlm2* in triggering plant defense response, DEGs associated with the resistance response at “Incomp” were identified by comparing “Incomp” vs. “Basal.” In total, 1, 016 up-regulated and 332 down-regulated DEGs were identified (Supplementary Table S3). To visualize the transcriptional alteration of these DEGs at different defense response levels ranging from “Basal,” “Comp” to “Incomp,” expression levels of DEGs were quantified through comparison with the mock-inoculated “Westar” control and translated into hierarchically clustering heatmap in three groups (Figure 2B). The expression profile amplitudes were determined as the average number of log2-fold change values. The heatmap showed an obvious increasing trend for up-regulated DEGs and a decreasing trend for down-regulated DEGs from “Basal” to “Comp” and to “Incomp.” In spite of the compatible interaction in “Comp,” *LepR3* and *Rlm2* triggered the defense transcriptome in “Comp” to a lesser extent compared to “Incomp.” The pairwise correlation index was also calculated among three groups to analyze the similarities of the expression profiles (Figure 2D,E). Correlation analysis showed both expression patterns of defense transcriptome for up-regulation and down-regulation at the “Incomp” group are closer to that at “Comp” than “Basal.” The up-regulated and down-regulated DEGs were also expressed in six individual inoculation within three groups (Figure 2C). The heatmap showed the similar trends of expression patterns for 1, 016 up-regulated and 332 down-regulated DEGs from “Westar” to compatible and to incompatible interaction on T35SLepR3 or TRlm2 inoculated by two isolates. Our results showed *LepR3* and *Rlm2*-mediated defense transcriptome has been induced in a limited scope at the absence of *AvrLm1* and *AvrLm2*, and is fully escalated to trigger resistance response upon recognition of *AvrLm1* and *AvrLm2*.

**FIGURE 3:**
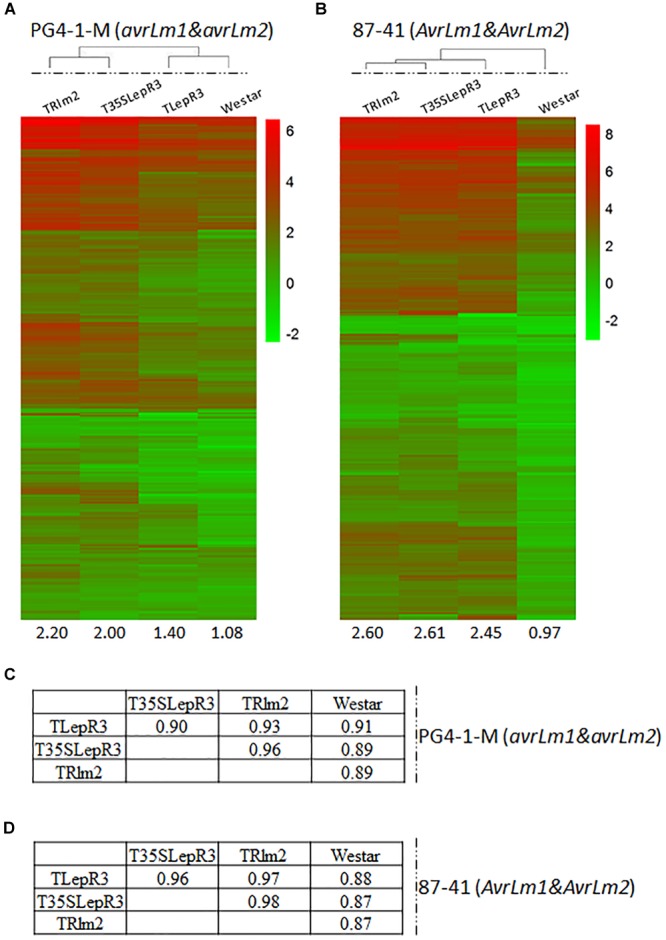
Correlation of *LepR3* and *Rlm2* expression with induction of 1,016 up-regulated DEGs during the compatible and incompatible interaction. **(A,B)** Heatmaps showing the expression profiles of identified 1,016 up-regulated DEGs on TRlm2, T35SLepR3, TLepR3, and “Westar” inoculated by *L. maculans* isolate PG4-1-M (*avrLm1* and *avrLm2*) **(A)** for the compatible interaction and 87-41 (*AvrLm1* and *AvrLm2*) **(B)** for the incompatible interaction. The heatmaps were generated based on log2-transformed fold change values comparing to the mock-inoculated “Westar.” The amplitudes of the profiles were indicated below the heatmaps. **(C,D)** Tables of pair-wise correlation for expression profiles of 1,016 up-regulated DEGs in **(C)** for the compatible and **(D)** incompatible interaction.

To further validate the regulatory role of *LepR3* and *Rlm2* on defense transcriptome during the compatible and incompatible interaction, the identified 1,016 up-regulated DEGs were expressed in TRlm2, T35SLepR3, and TLepR3 inoculated by PG4-1-M (*avrLm1* and *avrLm2*) for the compatible interaction and 87-41 (*AvrLm1* and *AvrLm2*) for the incompatible interaction. The visualization of expression patterns on the heatmap showed higher expression profiles on TRlm2, T35SLepR3 and TLepR3 compared to that on “Westar” inoculated with PG4-1-M, while the expression profiles displayed differentially induced levels that correlated with the induction levels of transgenic genes (Figure 3A). The correlation assay showed expression profiles induced on TRlm2 and T35SLepR3 were more similar compared to that induced on TLepR3 (Figure 3C). The variation on expression profiles due to differential expression of transgenic genes in TRlm2, T35SLepR3 and TLepR3 diminished during the incompatible interaction with 87-41 (Figure 3B), which is also suggested by the correlation index among all expression profiles (Figure 3D). The expression profiles were still more related between TRlm2 and T35SLepR3. In short, *LepR3* and *Rlm2* are able to promote plant defense-related transcriptome during both compatible and incompatible interaction, with the differences signified by the magnitude distinction of defense transcriptomic output.

### Cluster Analysis of 1,016 Up-Regulated DEGs Underlying *LepR3* and *Rlm2*-Mediated Resistance Response

**FIGURE 4:**
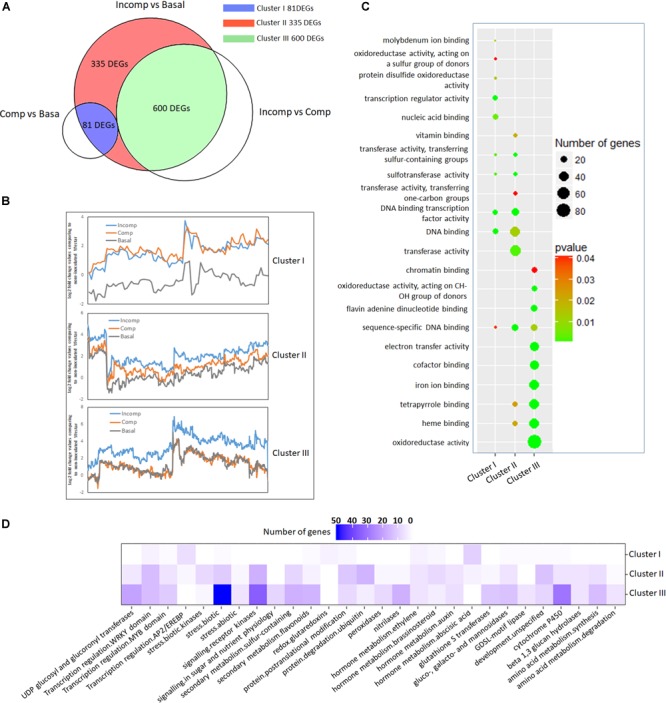
Cluster analysis of 1,016 up-regulated DEGs according to expression profiles and functional classification of each cluster. **(A)** Venn diagram showing the division of 1,016 up-regulated DEGs into three clusters through overlapping with DEGs identified from “Incomp” vs. “Comp” and “Comp” vs. “Basal” comparison. **(B)** Trend lines showing the gene expression profiles of DEGs in three clusters in “Basal,” “Comp,” and “Incomp.” The gene expression values were generated based on log2-transformed fold change values after comparing to the mock-inoculated “Westar.” **(C)** GO enrichment analysis. The molecular functions (MF) GO terms are represented in *y*-axis for each cluster. The top ten GO terms with a significant *p*-value (<0.05) for each cluster are shown. **(D)** Pathway analysis of DEGs. Pathway enrichment of each cluster DEGs was performed via the Mercator pipeline (http://www.plabipd.de/portal/mercator-sequence-annotation). The numbers of genes from each cluster DEGs in MapMan bins are translated into a gradation of color.

To dissect the gene expression network underlying *LepR3* and *Rlm2-*mediated resistance response, we classified 1,016 identified up-regulated DEGs according to their expression patterns in “Basal,” “Comp,” and “Incomp” groups. In this study, we also identified 811 up-regulated DEGs by comparing “Incomp” with “Comp” and 136 up-regulated DEGs by comparing “Comp” with “Basal” (Supplementary Table S3). Both of them largely overlapped with previously identified 1,016 DEGs by comparing “Incomp” with “Basal.” Therefore, 1,016 DEGs were separated into three clusters, including “Cluster I” DEGs overlapping with that identified in the “Comp” vs. “Basal” comparison, “Cluster III” DEGs overlapping with that identified in the “Incomp” vs. “Comp” comparison, and “Cluster II” DEGs sharing no overlapping (Figure 4A). Observation of expression patterns in the trend lines for three clusters’ DEGs showed that most “Cluster I” DEGs are suppressed at “Basal” and then expressed at the similar levels between “Comp” and “Incomp” (Figure 4B). “Cluster II” DEGs showed gradually induced expression levels from “Comp” to “Incomp,” which means the gene expression has been triggered by *LepR3* and *Rlm2* and further elevated with recognition of *AvrLm1* and *AvrLm2*. “Cluster III” DEGs showed no different expression patterns between “Basal” and “Comp” but solely induced at “Incomp” due to the *LepR3-AvrLm1* and *Rlm2-AvrLm2* recognition specificities.

To reveal the specific functions of three clusters’ DEGs, gene ontology (GO) enrichment in molecular function (MF) was performed (Figure 4C). The enrichment showed that most of “Cluster I” DEGs contained transcription factor activity. In “Cluster II” DEGs, the prominent GO terms included transferase activity, transcription factor activity, heme binding activity, and tetrapyrrole binding activity. “Cluster III” DEGs are mainly enriched in GO terms like oxidoreductase activity, heme binding activity, tetrapyrrole binding activity, iron ion binding activity, and co-factor binding activity, suggesting the involvement in the cellular oxidation-reduction reaction.

**FIGURE 5:**
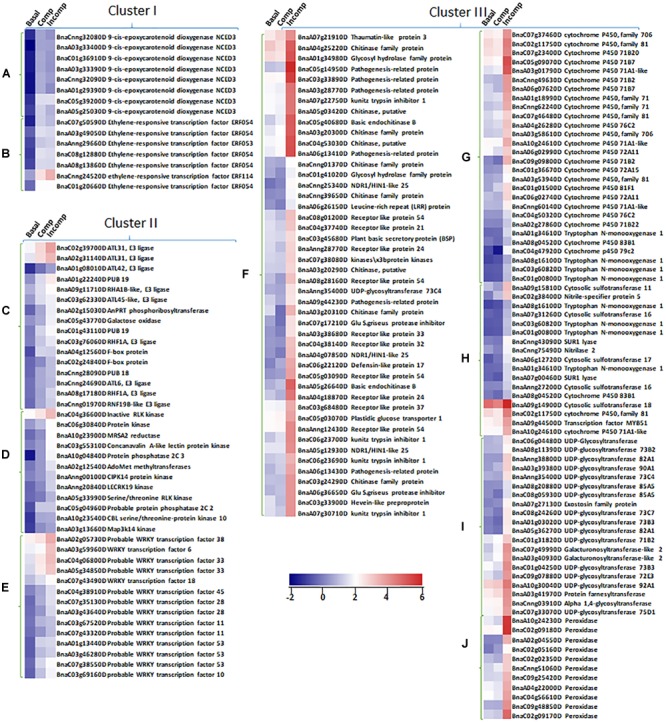
Expression profiles of up-regulated DEGs identified in unique biological pathways from “Cluster I,” “Cluster II,” and “Cluster III” in “Basal,” “Comp,” and “Incomp.” The heatmaps show the expression of abscisic acid (ABA) synthesis genes 9-*cis*-epoxycarotenoid dioxygenase NCED3 **(A)** and ethylene-responsive transcription factors ERFs **(B)** in “Cluster I,” the expression of genes in pathways of protein ubiquitination **(C)**, protein posttranslational modification **(D)**, and WRKY transcription factor family **(E)** in “Cluster II,” and the expression of genes in pathways of biotic stress response **(F)**, cytochrome P450 **(G)**, sulfur-containing secondary metabolism **(H)**, UDP glucosyl and glucoronyl transferases **(I)** and peroxidases **(J)**. The heatmaps were generated based on log2-transformed fold change values in comparison with the mock-inoculated “Westar.”

To identify biological pathways affiliated with DEGs at each cluster, enrichment was conducted using the MapMan program. The results showed that “Cluster I” DEGs were largely enriched in abscisic acid (ABA) hormone metabolism and ethylene-responsive transcription factors (Figure 4D, 5 and Supplementary Table S4), consistent with the MF GO enrichment of transcription factor activity. Receptor kinases for signaling transduction is the prominently enriched pathway for both “Cluster II” and “Cluster III” DEGs. This is in agreement with the previous investigation showing the critical roles of extracellular membrane RLPs and RLKs in the perception of fungal invasion and signaling transduction in *B. napus* non-host and race-specific resistance against *L. maculans* (Becker et al., 2017). Other leading pathways in “Cluster II” include protein ubiquitination, protein posttranslational modification, and WRKY transcriptional regulation (Figure 4D, 5 and Supplementary Table S4), in tandem with rich transferase activity that maybe involved in posttranslational modification and transcription factor activity during MF GO enrichment. The “Cluster III” DEGs are mainly enriched in pathways involved in plant defense-related physiological processes including response to biotic stress like deployment of pathogenesis-related proteins chitinases and kunitz trypsin inhibitors, cytochrome P450, sulfur-containing secondary metabolism, UDP glucosyl and glucoronyl transferases and peroxidases (Figure 4D, 5 and Supplementary Table S4). MF GO term enrichment suggests that these processes may require the participation of oxidation-reduction reactions.

### *LepR3* and *Rlm2* Regulate Abscisic Acid (ABA) Production Independent of Reaction to *L. maculans* Infection

**FIGURE 6:**
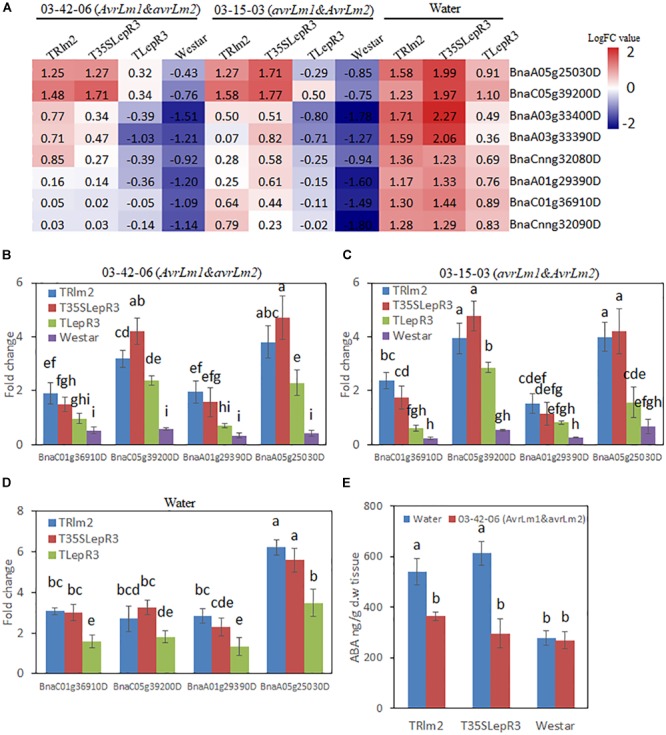
Characterization of the effect of *LepR3* and *Rlm2* on abscisic acid (ABA) production in *B. napus*. **(A)** The expression patterns of *B. napus* NCED3 genes identified in “Cluster I” on TRlm2, T35SLepR3, TLepR3, and “Westar” inoculated by 03-42-06 (*AvrLm1* and *avrLm2*), 03-15-03 (*avrLm1* and *AvrLm2*) or water at 4 dpi were displayed in the heatmap. The heatmap was generated based on log2-transformed fold change values comparing to the mock-inoculated “Westar.” **(B,D)** Validation of the expression patterns for selected NCED3 genes using RT-qPCR. Four NCED3 genes (BnaC01g36910D, BnaC05g39200D, BnaA01g29390D, and BnaA05g25030D) with the highest mapping reads in RNA-seq analysis were selected for the RT-qPCR analysis to test the expressional fold change compared to mock-inoculated “Westar” on TRlm2, T35SLepR3, TLepR3, and Westar inoculated by 03-42-06 (*AvrLm1* and *avrLm2*) **(B)**, 03-15-03 (*avrLm1* and *AvrLm2*) **(C)** or water **(D)** at 4 dpi. Error bars mean standard deviation based on data collected on three replicates in three independent experiments. Different letters indicate significant differences from each other, α = 0.05 (ANOVA). **(E)** Liquid chromatography-tandem mass spectrometry (LC-MS) analysis of ABA levels on TRlm2 and T35SLepR3 inoculated by 03-42-06 (*AvrLm1* and *avrLm2*) and water at 4 dpi. Values represent means ± standard errors from three independent experiments. Different letters indicate statistically significant differences, α = 0.05 (ANOVA).

**FIGURE 7:**
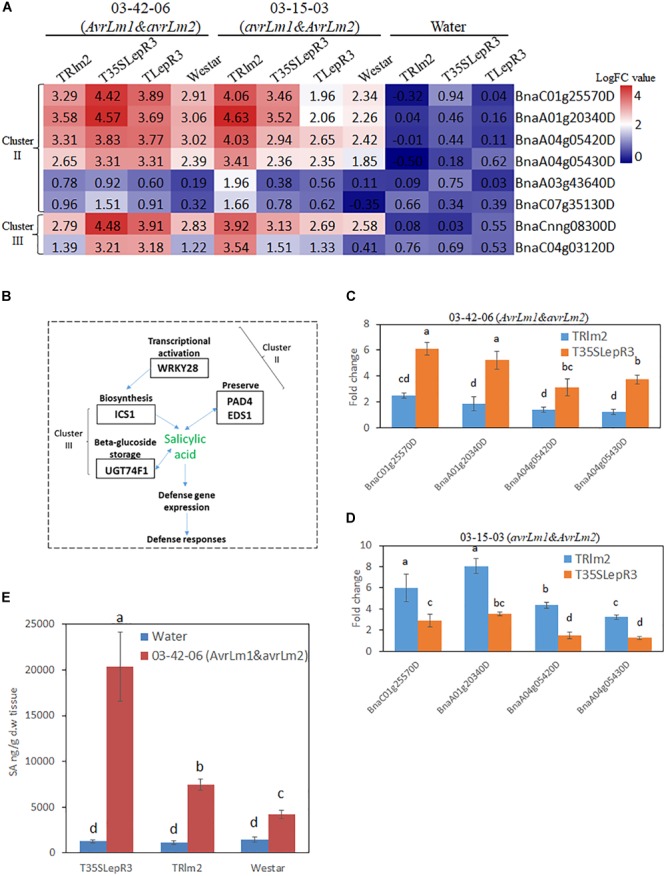
Regulation of salicylic acid (SA) synthesis by *LepR3* and *Rlm2* during the compatible and incompatible interaction. **(A)** The expression patterns of *B. napus* genes involved in SA biosynthesis identified in “Cluster II” and “Cluster III” on TRlm2, T35SLepR3, TLepR3, and “Westar” inoculated by 03-42-06 (*AvrLm1* and *avrLm2*), 03-15-03 (*avrLm1* and *AvrLm2*) or water at 4 dpi were displayed in the heatmap. The heatmap was generated based on log2-transformed fold change values comparing to the mock-inoculated “Westar.” **(B)** Overview of identified “Cluster II” and “Cluster III” DEGs participating in SA biosynthesis. **(C,D)** Validation of induction levels for selected genes using RT-qPCR. Two *PAD4* (BnaA04g05430D and BnaA04g05420D) and *EDS1* (BnaA01g20340D and BnaC01g25570D) genes were selected for the RT-qPCR analysis to test the induced fold change on TRlm2 and T35SLepR3 compared to “Westar” inoculated by 03-42-06 (*AvrLm1* and *avrLm2*) **(C)** and 03-15-03 (*avrLm1* and *AvrLm2*) **(D)** at 4 dpi. Error bars mean standard deviation based on data collected on three replicates in three independent experiments. Different letters indicate significant differences from each other, α = 0.05 (ANOVA). **(E)** Liquid chromatography-tandem mass spectrometry (LC-MS) analysis of SA levels on TRlm2 and T35SLepR3 inoculated by 03-42-06 (*AvrLm1* and *avrLm2*) and water at 4 dpi. Values represent means ± standard errors from three independent experiments. Different letters indicate statistically significant differences, α = 0.05 (ANOVA).

To further explore how *LepR3* and *Rlm2* modulate the ABA production in *B. napus*, the expression patterns of identified “Cluster I” NCED3 nine-*cis*-epoxycarotenoid dioxygenase 3 genes in three individual transgenic lines and “Westar” inoculated by *L. maculans* isolates (03-42-06 and 03-15-03) and water were displayed in the heatmap (Figure 6A). Surprisingly, NCED3 genes showed induction in TRlm2, T35SLepR3, and TLepR3 compared to “Westar” under the water treatment, with higher expressional levels observed in TRlm2 and T35SLepR3 compared to TLepR3. Inoculation of 03-42-06 (*AvrLm1* and *avrLm2*) or 03-15-03 (*avrLm1* and *AvrLm2*), however, down-regulated the expression patterns of NCED3 genes in all *B. napus* lines, regardless of compatible or incompatible interaction. To validate the RNA-seq analysis, RT-qPCR was conducted to assay the expression of four selected NCED3 genes with the highest counting reads in the RNA-seq analysis (Figure 6B–D). The results showed expression profiles of four genes in TRlm2, T35SLepR3, TLepR3, and “Westar” inoculated with 03-42-06 (Figure 6B), 03-15-03 (Figure 7C) or water (Figure 6D) were consistent with the RNA-seq analysis. Using liquid chromatography-mass spectrometry (LC-MS), the simultaneous ABA levels of TRlm2, T35SLepR3 and “Westar” inoculated by 03-42-06 or water were measured at 4 dpi (Figure 6E). In line with the gene expression data, TRlm2 and T35SLepR3 accumulated higher ABA than “Westar” under the water treatment. Inoculation of 03-42-06 reduced the ABA contents in both transgenic lines to the similar level in “Westar,” which nevertheless showed no change in ABA contents between mock and 03-42-06 inoculation. Therefore, it seems that *LepR3* and *Rlm2* as receptors acting at the apoplastic space may play roles in stress response beyond the defense against the *L. maculans* infection.

### *LepR3* and *Rlm2* Modulate the Salicylic Acid (SA) Production in Response to *L. maculans* Infection During Compatible and Incompatible Interaction

DEGs in “Cluster II” are mainly families involved in signal transduction in response to biotic stress, including WRKY transcriptional factors identified as a major group of DEGs. In particular, *WRKY 28* (BnaA03g43640D and BnaC07g35130D) homolog has been shown as a transcriptional activator of SA synthesis enzyme isochorismate synthase 1 (*ICS1*) in *Arabidopsis* (van Verk et al., 2011). Accordingly, another four SA signal regulators including two enhanced disease susceptibility (*EDS1*) genes (BnaC01g25570D and BnaA01g20340D) and two phytoalexin deficient 4 (*PAD4*) genes (BnaA04g05420D and BnaA04g05430D) that function in the alternative route to facilitate the SA preserve were identified in “Cluster II” (Supplementary Table S4). In contrast, *ICS1* (BnaCnng08300D) and UDP-glycosyltransferase 74F1 (UGT74F1) (BnaC04g03120D) catalyzing SA-glucose conjugation for storage were found in “Cluster III.” The gene expression patterns for individual inoculation on the heatmap (Figure 7A,B) showed *EDS1, PAD4*, and *WRKY28* were induced during the compatible interaction on TRlm2 and T35SLepR3 compared to “Westar” and transcriptionally culminated during the incompatible interaction. Moreover, RT-qPCR was performed to validate the expression profile for *EDS1* and *PAD4* genes, which showed these genes were significantly induced on TRlm2 and T35SLepR3 during the compatible interaction compared to “Westar” and further transcriptionally enhanced during the incompatible interaction (Figure 7C,D). Simultaneous measurement of SA content by LC-MS on TRlm2, T35SLepR3 and “Westar” inoculated by 03-42-06 (*AvrLm1* and *avrLm2*) and water showed that, in contrast to ABA production, the introduction of *Rlm2* and *LepR3* in *B. napus* has no effect on SA production without *L. maculans* infection (Figure 7E). However, inoculation of 03-42-06 on TRlm2 that caused compatible interaction, produced 1.8-time higher SA compared to “Westar” and inoculation on T35SLepR3 for incompatible interaction resulted in 4.8-time higher SA production. These results suggested that the SA-mediated defense signal network could be induced by *LepR3* and *Rlm2* during the compatible interaction and magnified to the maximum following the recognition of the *Avr* gene products for the incompatible interaction.

### Hydrogen Peroxide Production and Peroxidase Expression Were Exclusively Activated by *LepR3* and *Rlm2* During Resistance Response to the *L. maculans* Infection

**FIGURE 8:**
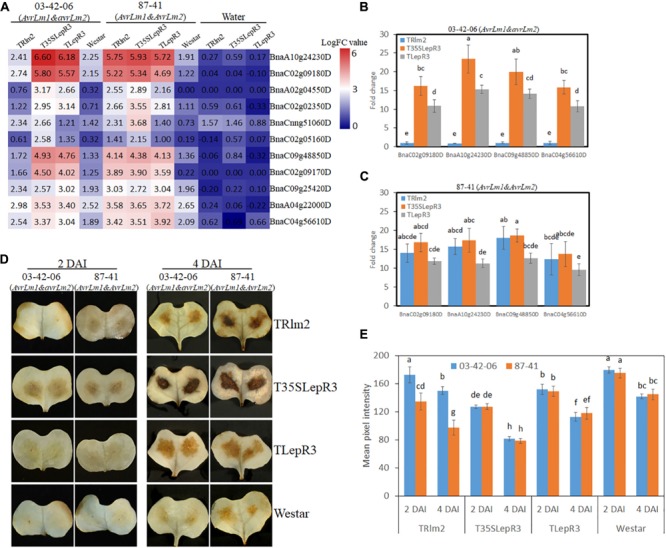
*LepR3* and *Rlm2* significantly enhance peroxidase expression and hydrogen peroxide (H_2_O_2_) production during the incompatible interaction. **(A)** The expression patterns of *B. napus* peroxidase genes identified in “Cluster III” on TRlm2, T35SLepR3, TLepR3, and “Westar” inoculated by 03-42-06 (*AvrLm1* and *avrLm2*), 87-41 (*AvrLm1* and *AvrLm2*) or water at 4 dpi were displayed in the heatmap. The heatmap was generated based on log2-transformed fold change values comparing to the mock-inoculated “Westar.” **(B,C)** Validation of induction levels for selected genes using RT-qPCR. Four peroxidase genes (BnaC02g09180D, BnaA10g24230D, BnaC09g48850D, and BnaC04g56610D) with the highest mapping reads in the RNA-seq analysis were selected to test the induction levels compared to “Westar” on TRlm2, T35SLepR3, and TLepR3 inoculated by 03-42-06 (*AvrLm1* and *avrLm2*) **(B)** and 87-41 (*AvrLm1* and *AvrLm2*) **(C)** at 4 dpi. Error bars mean standard deviation based on data collected on three replicates in three independent experiments. Different letters indicate significant differences from each other, α = 0.05 (ANOVA). **(D)** Cotyledons of *B. napus* seedlings (TRlm2, T35SLepR3, TLepR3, and “Westar”) were inoculated by infiltration of the spore suspension of indicated isolates 03-42-06 (*AvrLm1* and *avrLm2*) and 87-41 (*AvrLm1* and *AvrLm2*) for 2 and 4 days, and H_2_O_2_ was detected *in situ* by diaminobenzidine (DAB) staining. **(E)** Bar charts showing mean pixel intensity of DAB staining on *B. napus* lines at 2 and 4 dpi. Values represent means ± standard errors from three independent experiments with 10 cotyledons collected for each experiment. Different letters indicate statistically significant differences, α = 0.05 (ANOVA).

One major function of DEGs in “Cluster III” is to promote the cellular oxidation-reduction reaction and causes the hypersensitive response (HR)-related physiological reactions. Enhanced accumulation of reactive oxygen species (ROS) at the infection sites is one hallmark of HR resulting from the recognition of pathogen *Avr* genes (Torres et al., 2006). In our RNA-seq analysis, 11 peroxidase genes that are rapidly responsive to extracellular hydrogen peroxide (H_2_O_2_) accumulation and involved in ROS generation for cell wall modification and resistance response were identified in “Cluster III” (Shigeto and Tsutsumi, 2016). The expressional levels of peroxidase genes on TRlm2, T35SLepR3, TLepR3, and “Westar” inoculated by 03-42-06 (*AvrLm1* and *avrLm2*) and 87-41 (*AvrLm1* and *AvrLm2*) for the incompatible interaction were visualized in the heatmap (Figure 8A). Most of them were significantly induced on T35SLepR3 and TLepR3 inoculated by 03-42-06 for invoking the incompatible interaction compared to inoculation on TRlm2 and “Westar,” similar to the expression pattern of “Cluster III” DEGs at “Basal,” “Comp,” and “Incomp.” Inoculation of 87-41 on TRlm2 and T35SLepR3 induced the similar expression patterns for peroxidase genes. Validation of four selected genes using RT-qPCR on TRlm2, T35SLepR3, and TLepR3 showed the significant induction during the incompatible interaction compared to “Westar” (Figure 8B,C), while inoculation of 03-42-06 on TRlm2 did not cause significant variation. Moreover, higher gene expression was always induced on T35SLepR3 compared to TLepR3 in both RNA-seq and RT-qPCR analysis. These results were consistent with the *in situ* detection of H_2_O_2_ at the infection sites at 2 and 4 dpi on the same *B. napus* lines, which showed significantly intensified H_2_O_2_ production due to the resistance response on three transgenic lines (Figure 8D,E). Notably, higher H_2_O_2_ was produced on T35SLepR3 compared to TLepR3 during the incompatible interaction with both 03-41-06 and 87-41 isolates, while stronger H_2_0_2_ burst was always observed at 4 dpi compared to 2 dpi. In short, their results indicate that the scale of resistance response is proportional to the induction timing and level of *LepR3* and *Rlm2*.

## Discussion

Recently, RNA-seq has been extensively used to decipher the transcriptomic reprogramming underpinning *B. napus* defense response against *L. maculans* infection (Lowe et al., 2014; Haddadi et al., 2016; Becker et al., 2017). However, the transcriptomic comparison made between resistant and susceptible cultivars differing at the genetic background biases the analysis. In this study, we created transgenic lines of resistance genes *LepR3* and *Rlm2* in the susceptible cultivar “Westar” to reveal the *R* gene-mediated defense machinery based on the transcriptomic comparison (Figure 1). Four *L. maculans* isolates with different (*AvrLm1* and *AvrLm2*) patterns were used for inoculation to distinguish the defense response mediated by *LepR3* and *Rlm2* in comparison with “Westar.”

Contrary to ETI-mediated defense response ramifying from the intracellular *R*-*Avr* gene recognition and quickly activating downstream cellular responses to cause plant resistance (Dodds and Rathjen, 2010), defense response initiated by the surface-localized receptor-like proteins (RLPs) recognizing pathogen effectors at the plant apoplastic space is termed as effector-triggered defense (ETD) (Stotz et al., 2014), characterized by relatively slower downstream cellular responses. Although surface-localized RLPs triggering resistance against apoplastic fungal pathogens have been cloned in several crops (Stotz et al., 2014), the acting machinery of regulating the gene expression network underlying the resistance remains elusive. Both LepR3 and Rlm2 bind the leucine-rich repeat receptor-like kinase (LRR-RLK) SOBIR1 to form the complex for triggering the downstream defense signals for plant resistance (Ghanbarnia et al., 2015; Ma and Borhan, 2015). In addition, SOBIR1 is also able to physically interact with cell surfaced localized RLPs recognizing fungal PAMPs like fungal endopolygalacturonases to trigger PTI in *Arabidopsis* (Zhang et al., 2014). This may implicate the signal pathways leading PTI and ETD in the same plant may overlap but differ at the timing and magnitudes. Transcriptomic profiling has demonstrated that plant defense pathways between compatible and incompatible interactions are shared but vary at the output scale (Tao et al., 2003, Thomma et al., 2011).

In this study, we intended to investigate the role of *LepR3* and *Rlm2* in regulating the defense signal output during compatible and incompatible interaction. Through the classification of 03-42-06 (*AvrLm1* and *avrLm2*) and 03-15-03 (*avrLm1* and *AvrLm2*) inoculation on T35SLepR3, TRlm2 and “Westar” into three groups, defense response during the compatible interaction on T35SLepR3 and TRlm2 termed as “Comp” can also be transcriptionally measured (Supplementary Figure S3). The gradually changed scale of gene expression and closer-related gene expression profiles between “Comp” and “Incomp” suggest that *LepR3* and *Rlm2* are able to promote the defense response without *AvrLm1* and *AvrLm2* recognition and may target at other pathogen-associated components (Figure 2). In spite of high sequence and structure similarities shared between LepR3 and Rlm2, AvrLm1, and AvrLm2 do not share any sequence similarity (Gout et al., 2006; Ghanbarnia et al., 2015). It has been proposed that biologically diversified effectors can target the collective set of plant proteins as joint points of various cellular mechanisms and are crucial for effective defense response (Mukhtar et al., 2011). Conversely, *LepR3* and *Rlm2* may monitor multiple fungal targets that contribute to the *L. maculans* virulence. PG4-1-M (*avrLm1* and *avrLm2*) inoculation on TRlm2, T35LepR3, and TLepR3 can also induce higher defense transcriptome on *B. napus* compared to “Westar,” in correlation with the expression level of *Rlm2* or *LepR3* (Figure 3A,C). Indeed, the tomato basal defense response against bacterial and fungal pathogens is enhanced when overexpressing the tomato *R* gene *Pto* that confers race-specific resistance to *Pseudomonas syringae* containing the type III effector protein AvrPto (Mysore et al., 2003). Taken together, our results may implicate the possible role of *LepR3* or *Rlm2* in invoking the basal defense during the compatible interaction.

The functional classification of 1,016 up-regulated DEGs according to the expression patterns at “Basal,” “Comp” and “Incomp” gives insight into how *LepR3* and *Rlm2* orchestrate the gene expression network in response to biotic/abiotic stresses. The famous role of ABA in regulating the crosstalk of biotic and abiotic stress responses via the interaction with other hormone signaling pathways has been widely studied (Lee and Luan, 2012). Although slight induction of NCED3 genes was observed during the compatible and incompatible interaction on T35SLepR3 and TRlm2 compared to “Westar,” this may not be significant enough to cause the ABA level change detected in the LC-MS assay (Figure 6). This is in line with the finding in (Šašek et al., 2012) that the ABA level remained unchanged during the early resistance response triggered by *Rlm1-AvrLm1* recognition specificity compared to “Westar.” However, the unexpected role of *LepR3* and *Rlm2* in regulating ABA synthesis without *L. maculans* infection may suggest these RLPs are involved in abiotic stress response (Figure 6). Recently, an NBS-LRR disease resistance gene *AhRRS5* in peanut has been shown to respond transcriptionally to cold and drought conditions as well as ethylene and ABA treatment (Zhang et al., 2017). This is correlating with our finding showing the concomitant expression pattern of NCED3 genes and apetala2/ethylene responsive factors (AP2/ERFs) in “Cluster I” (Figure 5), as the reciprocal relation between AP2/ERF regulation and ABA biosynthesis in coping with biotic and abiotic stresses has been previously revealed (Song et al., 2005; Lin et al., 2007). Inoculation on T35SLepR3 and TRlm2 counteracted the ABA change (Figure 6E), coinciding with induced SA production compared to “Westar” (Figure 7E), may suggest an antagonistic interconnection between the abiotic stress responsive ABA-mediated signaling and disease defense responsive SA-mediated signaling under the regulation of *LepR3* and *Rlm2*. The negative regulatory effect of pathogen or host-derived ABA on SA-mediated systemic acquired resistance (SAR) has been described in several bacterial and fungal-plant pathosystems (Yasuda et al., 2008; de Torres Zabala et al., 2009; Jiang et al., 2010). A recent RNA-seq analysis has revealed the clustered expression of genes responsible for ABA synthesis, involved in suppression of *B. napus* defense response, in *L. maculans* genome (Darma et al., 2019). By de-activating the ABA synthesis and activating the SA synthesis in response to *L. maculans* infection, LepR3 and Rlm2 may orchestrate a fine-turn hormonal dynamic to optimize the defense response.

“Cluster II” DEGs are mainly involved in signal transduction to mediate the stress/defense response during both compatible and incompatible interaction (Figure 4, 5). Previous studies have indicated the regulatory role of WRKY transcription factors (TFs) in hormone responsive pathways during the compatible interaction between *B. napus* and *L. maculans* (Yang et al., 2009). In this study, WRKY28 as the transcriptional activator of SA biosynthesis gene *ICS1* and PD4/EDS1 as regulatory components promoting SA preserve acting independently of the *ICS1* pathway (Cui et al., 2017) are simultaneously identified in “Cluster II” (Figure 7A,B). As SA is a major plant hormone to activate both PTI/ETI systems, slightly higher SA production detected on TRlm2 inoculated by 03-42-06 (*AvrLm1* and *avrLm2*) compared to “Westar” indicates *LepR3* or *Rlm2* is able to promote the defense response via activation of hormone signal pathways during the compatible interaction (Figure 7E). This confirms the observation of higher expression profiles of defense transcriptome on TRlm2, T35SLepR3, and TLepR3 compared to “Westar” inoculated by PG4-1-M (*avrLm1* and *avrLm2*) (Figure 3A,C). WRKY45 is another identified WRKY TF that has been showed to be physically targeted by the *Panicle blast 1* (*Pb1*) resistance gene to mediate the broad-spectrum resistance against *M. oryzae* in rice (Inoue et al., 2013), through activation of downstream defense-related genes such as cytochrome P450s, peroxidases, and chitinases (Nakayama et al., 2013). RING-finger and U-box domain-containing E3 ligase genes are another “Cluster II” DEG group and involved in posttranslational protein modification for defense signal activation during both compatible and incompatible interaction. *ATL6* and *ATL31* homologs are induced with the treatment of PAMP elicitor flg22 and regulate the cross-talk of plant nutrient status and defense response in *Arabidopsis* (Maekawa et al., 2012), while *ATL3* homolog was induced during the *cf-9* mediated resistance in tomato against *Cladosporium fulvum* (Durrant et al., 2000). Taken together, we may infer that *LepR3* and *Rlm2* can activate the signal transduction pathways, shared by PTI/ETI systems in response to the biotic stress, without recognition of *AvrLm1* or *AvrLm2*.

A large part of DEGs identified in “Cluster III” include degradation enzymes, protease inhibitors and redox-related enzymes for ROS burst and secondary metabolism homeostasis that contribute to contain the fungal growth and trigger the hypersensitive response (HR) (Figure 5). In Šašek et al. (2012), enhanced H_2_O_2_ production is triggered following recognition of avirulent *L. maculans* isolate in *B. napus*. Our study showed the exclusive H_2_O_2_ burst at the infection sites and the consequential induction of peroxidase genes during the incompatible interaction are regulated by the infection timing and *R* gene induction level, as more significantly intensified DAB staining is observed in T35SLepR3 than TLepR3 at 2 and 4 dpi (Figure 8). This indicates that higher expression of *LepR3* is able to more rapidly promote defense transcriptome and cause earlier resistance response. Another major identified group of enzymes involved in cellular redox potential are P450 hemoproteins (Figure 5), which facilitate the biosynthesis of anti-microbial secondary metabolites, like glucosinolate and phytoalexin in the Brassicaceae family through the redox reactions, and thus play important roles in disease resistance (Del Carmen Martínez-Ballesta et al., 2013; Jun et al., 2015). Detection of tryptophan N-monooxygenase 1 and cytosolic sulfotransferase that facilitate the biosynthesis of glucosinolate and UDP-glycosyltransferases (UGTs) that are key for the homeostasis maintenance of plant secondary metabolites further suggests that *LepR3* and *Rlm2* regulate the deployment of chemical protection during the incompatible interaction (Figure 5). Identification of chitinases shown to target at *L. maculans* at the apoplastic space of pre-symptom stage cotyledons in previous studies during the incompatible interaction (Legrand et al., 1987; Profotová et al., 2007) and kunitz trypsin inhibitors that degrade the peptidases secreted by *L. maculans* during the early infection stage (Sonah et al., 2016) implicates the direct deployment of enzymatic force by *LepR3* and *Rlm2* to fend off *L. maculans* during *in planta* invasion (Figure 5).

Through RNA-seq based transcriptomic profiling and comparison, we delineate a hierarchical gene expression network orchestrated by *LepR3* and *Rlm2* to mobilize plant defense response against *L. maculans* infection. The biotic stress/ defense-responsive signal transduction pathways shared by PTI/ETI systems are activated by *LepR3* and *Rlm2* during the compatible interaction and magnified via recognition of *AvrLm1* and *AvrLm2*, finally causing deployment of chemicals and enzymes to cause HR response. The future studies will focus on functionally characterizing the key components of signal transduction and metabolic pathways identified in this study and elaborating the interaction machinery via biochemistry and cell biology approaches.

## Data Availability

The datasets generated for this study can be found in NCBI BioProject, PRJNA378851.

## Author Contributions

TZ and GL designed the experiments and wrote the manuscript. TZ, ZL, and PT performed the experiments. TZ, WX, and AH analyzed the data. TZ, BA, FD, PM, RD, and GL revised the manuscript.

## Conflict of Interest Statement

The authors declare that the research was conducted in the absence of any commercial or financial relationships that could be construed as a potential conflict of interest.

## Footnotes

1 http://www.cambia.org

2 http://www.ncbi.nlm.nih.gov/sra/

3 http://revigo.irb.hr

4 http://mapman.gabipd.org/web/guest/app/mercator

## References

[B1] Anders S. Pyl P. T. Huber W. (2015). HTSeq-a python framework to work with high-throughput sequencing data. *Bioinformatics* 31 166–169. 10.1093/bioinformatics/btu638 25260700 PMC4287950

[B2] Balesdent M. H. Fudal I. Ollivier B. Bally P. Grandaubert J. Eber F. (2013). The dispensable chromosome of *Leptosphaeria maculans* shelters an effector gene conferring avirulence towards Brassica rapa. *New Phytol.* 198 887–898. 10.1111/nph.12178 23406519

[B3] Becker M. G. Zhang X. Walker P. L. Wan J. C. Millar J. L. Khan D. (2017). Transcriptome analysis of the *Brassica napus–Leptosphaeria maculans* pathosystem identifies receptor, signaling and structural genes underlying plant resistance. *Plant J.* 90 573–586. 10.1111/tpj.13514 28222234

[B4] Bouaid A. Martinez M. Aracil J. (2009). Production of biodiesel from bioethanol and *Brassica carinata* oil: oxidation stability study. *Bioresour. Technol.* 100 2234–2239. 10.1016/j.biortech.2008.10.045 19091551

[B5] Cook D. E. Mesarich C. H. Thomma B. P. (2015). Understanding plant immunity as a surveillance system to detect invasion. *Annu. Rev. Phytopathol.* 53 541–563. 10.1146/annurev-phyto-080614-120114 26047564

[B6] Cui H. Gobbato E. Kracher B. Qiu J. Bautor J. Parker J. E. (2017). A core function of *EDS1* with *PAD4* is to protect the salicylic acid defense sector in *Arabidopsis* immunity. *New Phytol.* 213 1802–1817. 10.1111/nph.14302 27861989

[B7] Darma R. Lutz A. Elliott C. E. Idnurm A. (2019). Identification of a gene cluster for the synthesis of the plant hormone abscisic acid in the plant pathogen *Leptosphaeria maculans*. *Fungal Genet. Biol.* 130 62–71. 10.1016/j.fgb.2019.04.015 31034868

[B8] de Torres Zabala M. Bennett M. H. Truman W. H. Grant M. R. (2009). Antagonism between salicylic and abscisic acid reflects early host-pathogen conflict and moulds plant defence responses. *Plant J.* 59 375–386. 10.1111/j.1365-313X.2009.03875.x 19392690

[B9] Del Carmen Martínez-Ballesta M. Moreno D. A. Carvajal M. (2013). The physiological importance of glucosinolates on plant response to abiotic stress in *Brassica*. *Int. J. Mol. Sci.* 14 11607–11625. 10.3390/ijms140611607 23722664 PMC3709749

[B10] Delourme R. Chevre A. Brun H. Rouxel T. Balesdent M. Dias J. (2006). Major gene and polygenic resistance to *Leptosphaeria maculans* in oilseed rape (*Brassica napus*). *Eur. J. Plant Pathol.* 114 41–52. 10.1094/PD-90-0190 30786411

[B11] Dodds P. N. Rathjen J. P. (2010). Plant immunity: towards an integrated view of plant–pathogen interactions. *Nat. Rev. Genet.* 11:539. 10.1038/nrg2812 20585331

[B12] Durrant W. E. Rowland O. Piedras P. Hammond-Kosack K. E. Jones J. D. (2000). cDNA-AFLP reveals a striking overlap in race-specific resistance and wound response gene expression profiles. *Plant Cell* 12 963–977. 10852940 10.1105/tpc.12.6.963 PMC149096

[B13] Ecke W. Uzunova M. Weissleder K. (1995). Mapping the genome of rapeseed (*Brassica napus* L.). II. Localization of genes controlling erucic acid synthesis and seed oil content. *Theor. Appl. Genet.* 91 972–977. 10.1007/BF00223908 24169985

[B14] Falcon S. Gentleman R. (2006). Using GOstats to test gene lists for GO term association. *Bioinformatics* 23 257–258. 17098774 10.1093/bioinformatics/btl567

[B15] Fudal I. Ross S. Gout L. Blaise F. Kuhn M. Eckert M. (2007). Heterochromatin-like regions as ecological niches for avirulence genes in the *Leptosphaeria maculans* genome: map-based cloning of *AvrLm6*. *Mol. Plant Microbe Interact.* 20 459–470. 17427816 10.1094/MPMI-20-4-0459

[B16] Ghanbarnia K. Fudal I. Larkan N. J. Links M. G. Balesdent M. H. Profotova B. (2015). Rapid identification of the *Leptosphaeria maculans* avirulence gene *AvrLm2* using an intraspecific comparative genomics approach. *Mol. Plant Pathol.* 16 699–709. 10.1111/mpp.12228 25492575 PMC6638346

[B17] Gout L. Fudal I. Kuhn M. L. Blaise F. Eckert M. Cattolico L. (2006). Lost in the middle of nowhere: the *AvrLm1* avirulence gene of the dothideomycete *Leptosphaeria maculans*. *Mol. Microbiol.* 60 67–80. 16556221 10.1111/j.1365-2958.2006.05076.x

[B18] Hacquard S. Spaepen S. Garrido-Oter R. Schulze-Lefert P. (2017). Interplay between innate immunity and the plant microbiota. *Annu. Rev. Phytopathol.* 55 565–589. 10.1146/annurev-phyto-080516-035623 28645232

[B19] Haddadi P. Ma L. Wang H. Borhan M. H. (2016). Genome-wide transcriptomic analyses provide insights into the lifestyle transition and effector repertoire of *Leptosphaeria maculans* during the colonization of *Brassica napus* seedlings. *Mol. Plant Pathol.* 17 1196–1210. 10.1111/mpp.12356 26679637 PMC6638455

[B20] Howlett B. J. Idnurm A. Pedras M. S. C. (2001). *Leptosphaeria maculans*, the causal agent of blackleg disease of Brassicas. *Fungal Genet. Biol.* 33 1–14. 11407881 10.1006/fgbi.2001.1274

[B21] Huang Y. J. Evans N. Li Z. Q. Eckert M. Chèvre A. M. Renard M. (2006). Temperature and leaf wetness duration affect phenotypic expression of *Rlm6*-mediated resistance to *Leptosphaeria maculans* in *Brassica napus*. *New Phytol.* 170 129–141. 16539610 10.1111/j.1469-8137.2005.01651.x

[B22] Inoue H. Hayashi N. Matsushita A. Xinqiong L. Nakayama A. Sugano S. (2013). Blast resistance of CC-NB-LRR protein Pb1 is mediated by WRKY45 through protein-protein interaction. *Proc. Natl. Acad. Sci. U.S.A.* 110 9577–9582. 10.1073/pnas.1222155110 23696671 PMC3677490

[B23] Jiang C. J. Shimono M. Sugano S. Kojima M. Yazawa K. Yoshida R. (2010). Abscisic acid interacts antagonistically with salicylic acid signaling pathway in rice-*Magnaporthe grisea* interaction. *Mol. Plant Microbe Interact.* 23 791–798. 10.1094/MPMI-23-6-0791 20459318

[B24] Jones J. D. Dangl J. L. (2006). The plant immune system. *Nature* 444:323. 10.1038/nature05286 17108957

[B25] Jones J. T. Haegeman A. Danchin E. G. J. Gaur H. S. Helder J. Jones M. G. K. (2013). Top 10 plant-parasitic nematodes in molecular plant pathology. *Mol. Plant Pathol.* 14 946–961. 10.1111/mpp.12057 23809086 PMC6638764

[B26] Jun X. Wang X. Y. Guo W. Z. (2015). The cytochrome P450 superfamily: key players in plant development and defense. *J. Integ. Agric.* 14 1673–1686.

[B27] Kanno Y. Jikumaru Y. Hanada A. Nambara E. Abrams S. R. Kamiya Y. (2010). Comprehensive hormone profiling in developing *Arabidopsis* seeds: examination of the site of ABA biosynthesis, ABA transport and hormone interactions. *Plant Cell Physiol.* 51 1988–2001. 10.1093/pcp/pcq158 20959378

[B28] Langmead B. Salzberg S. L. (2012). Fast gapped-read alignment with Bowtie 2. *Nat. Methods* 9:357. 10.1038/nmeth.1923 22388286 PMC3322381

[B29] Larkan N. Lydiate D. Parkin I. Nelson M. Epp D. Cowling W. (2013). The *Brassica napus* blackleg resistance gene *LepR3* encodes a receptor-like protein triggered by the *Leptosphaeria maculans* effector *AvrLm1*. *New Phytol.* 197 595–605. 23206118 10.1111/nph.12043

[B30] Larkan N. J. Lydiate D. J. Yu F. Rimmer S. R. Borhan M. H. (2014). Co-localisation of the blackleg resistance genes *Rlm2* and *LepR3* on *Brassica napus* chromosome A10. *BMC Plant Biol.* 14:387. 10.1186/s12870-014-0387-z 25551287 PMC4302512

[B31] Larkan N. J. Ma L. Borhan M. H. (2015). The *Brassica napus* receptor-like protein RLM2 is encoded by a second allele of the *LepR3/Rlm2* blackleg resistance locus. *Plant Biotechnol. J.* 13 983–992. 10.1111/pbi.12341 25644479

[B32] Lee S. C. Luan S. (2012). ABA signal transduction at the crossroad of biotic and abiotic stress responses. *Plant Cell Environ.* 35 53–60. 10.1111/j.1365-3040.2011.02426.x 21923759

[B33] Legrand M. Kauffmann S. Geoffroy P. Fritig B. (1987). Biological function of pathogenesis-related proteins: four tobacco pathogenesis-related proteins are chitinases. *Proc. Natl. Acad. Sci. U.S.A.* 84 6750–6754. 16578819 10.1073/pnas.84.19.6750 PMC299162

[B34] Lin R. Zhao W. Meng X. Peng Y. L. (2007). Molecular cloning and characterization of a rice gene encoding AP2/EREBP-type transcription factor and its expression in response to infection with blast fungus and abiotic stresses. *Physiol. Mol. Plant Pathol.* 70 60–68.

[B35] Liu Z. Hammerlindl J. Keller W. Mcvetty P. B. Daayf F. Quiros C. F. (2011). MAM gene silencing leads to the induction of C3 and reduction of C4 and C5 side-chain aliphatic glucosinolates in *Brassica napus*. *Mol. Breed.* 27 467–478.

[B36] Livak K. J. Schmittgen T. D. (2001). Analysis of relative gene expression data using real-time quantitative PCR and the 2-ΔΔCT method. *Methods* 25 402–408. 11846609 10.1006/meth.2001.1262

[B37] Long Y. Wang Z. Sun Z. Fernando D. W. Mcvetty P. B. Li G. (2011). Identification of two blackleg resistance genes and fine mapping of one of these two genes in a *Brassica napus* canola cultivar ‘Surpass 400’. *Theor. Appl. Genet.* 122 1223–1231. 21258998 10.1007/s00122-010-1526-z

[B38] Love M. I. Huber W. Anders S. (2014). Moderated estimation of fold change and dispersion for RNA-seq data with DESeq2. *Genome Biol.* 15:550. 25516281 10.1186/s13059-014-0550-8 PMC4302049

[B39] Lowe R. G. Cassin A. Grandaubert J. Clark B. L. Van De Wouw A. P. Rouxel T. (2014). Genomes and transcriptomes of partners in plant-fungal-interactions between canola (*Brassica napus*) and two *Leptosphaeria* species. *PLoS One* 9:e103098. 10.1371/journal.pone.0103098 25068644 PMC4113356

[B40] Ma L. Borhan M. H. (2015). The receptor-like kinase SOBIR1 interacts with Brassica napus LepR3 and is required for Leptosphaeria maculans AvrLm1-triggered immunity. *Front. Plant Sci.* 6:933. 10.3389/fpls.2015.00933 26579176 PMC4625043

[B41] Maekawa S. Sato T. Asada Y. Yasuda S. Yoshida M. Chiba Y. (2012). The *Arabidopsis* ubiquitin ligases ATL31 and ATL6 control the defense response as well as the carbon/nitrogen response. *Plant Mol. Biol.* 79 217–227. 10.1007/s11103-012-9907-0 22481162

[B42] Mukhtar M. S. Carvunis A. R. Dreze M. Epple P. Steinbrenner J. Moore J. (2011). Independently evolved virulence effectors converge onto hubs in a plant immune system network. *Science* 333 596–601. 10.1126/science.1203659 21798943 PMC3170753

[B43] Mysore K. S. D’ascenzo M. D. He X. Martin G. B. (2003). Overexpression of the disease resistance gene *Pto* in tomato induces gene expression changes similar to immune responses in human and fruitfly. *Plant Physiol.* 132 1901–1912. 12913147 10.1104/pp.103.022731 PMC181276

[B44] Nakayama A. Fukushima S. Goto S. Matsushita A. Shimono M. Sugano S. (2013). Genome-wide identification of *WRKY45*-regulated genes that mediate benzothiadiazole-induced defense responses in rice. *BMC Plant Biol.* 13:150. 10.1186/1471-2229-13-150 24093634 PMC3850545

[B45] Parlange F. Daverdin G. Fudal I. Kuhn M. L. Balesdent M. H. Blaise F. (2009). *Leptosphaeria maculans* avirulence gene *AvrLm4-7* confers a dual recognition specificity by the *Rlm4* and *Rlm7* resistance genes of oilseed rape, and circumvents *Rlm4*-mediated recognition through a single amino acid change. *Mol. Microbiol.* 71 851–863. 10.1111/j.1365-2958.2008.06547.x 19170874

[B46] Patel R. K. Jain M. (2012). NGS QC Toolkit: a toolkit for quality control of next generation sequencing data. *PLoS One* 7:e30619. 10.1371/journal.pone.0030619 22312429 PMC3270013

[B47] Plissonneau C. Daverdin G. Ollivier B. Blaise F. Degrave A. Fudal I. (2016). A game of hide and seek between avirulence genes *AvrLm4-7* and *AvrLm3* in *Leptosphaeria maculans*. *New Phytol.* 209 1613–1624. 10.1111/nph.13736 26592855

[B48] Profotová B. Burketová L. Valentová O. (2007). Chitinase isozymes induced by TYMV and *Leptosphaeria maculans* during compatible and incompatible interaction with *Brassica napus*. *Biol. Plant.* 51 507–513.

[B49] Rimmer S. R. (2006). Resistance genes to *Leptosphaeria maculans* in *Brassica napus*. *Can. J. Plant Pathol.* 28 S288–S297.

[B50] Rouxel T. Grandaubert J. Hane J. K. Hoede C. Van De Wouw A. P. Couloux A. (2011). Effector diversification within compartments of the *Leptosphaeria maculans* genome affected by repeat-induced point mutations. *Nat. Commun.* 2:202. 10.1038/ncomms1189 21326234 PMC3105345

[B51] Šašek V. Nováková M. Jindøichová B. Bóka K. Valentová O. Burketová L. (2012). Recognition of avirulence gene *AvrLm1* from hemibiotrophic ascomycete *Leptosphaeria maculans* triggers salicylic acid and ethylene signaling in *Brassica napus*. *Mol. Plant Microbe Interact.* 25 1238–1250. 10.1094/MPMI-02-12-0033-R 22624662

[B52] Schneider C. A. Rasband W. S. Eliceiri K. W. (2012). NIH Image to ImageJ: 25 years of image analysis. *Nat. Methods* 9:671. 10.1038/nmeth.2089 PMC5554542 22930834

[B53] Shigeto J. Tsutsumi Y. (2016). Diverse functions and reactions of class III peroxidases. *New Phytol.* 209 1395–1402. 10.1111/nph.13738 26542837

[B54] Son S. H. Chitnis V. R. Liu A. Gao F. Nguyen T.-N. Ayele B. T. (2016). Abscisic acid metabolic genes of wheat (*Triticum aestivum* L.): identification and insights into their functionality in seed dormancy and dehydration tolerance. *Planta* 244 429–447. 10.1007/s00425-016-2518-2 27091738

[B55] Sonah H. Zhang X. Deshmukh R. K. Borhan M. H. Fernando W. D. Bélanger R. R. (2016). Comparative transcriptomic analysis of virulence factors in *Leptosphaeria maculans* during compatible and incompatible interactions with canola. *Front. Plant Sci.* 7:1784. 10.3389/fpls.2016.01784 27990146 PMC5131014

[B56] Song C.-P. Agarwal M. Ohta M. Guo Y. Halfter U. Wang P. (2005). Role of an Arabidopsis AP2/EREBP-type transcriptional repressor in abscisic acid and drought stress responses. *Plant Cell* 17 2384–2396. 15994908 10.1105/tpc.105.033043 PMC1182496

[B57] Stotz H. U. Mitrousia G. K. De Wit P. J. Fitt B. D. (2014). Effector-triggered defence against apoplastic fungal pathogens. *Trends Plant Sci.* 19 491–500. 10.1016/j.tplants.2014.04.009 24856287 PMC4123193

[B58] Tao Y. Xie Z. Chen W. Glazebrook J. Chang H. S. Han B. (2003). Quantitative nature of *Arabidopsis* responses during compatible and incompatible interactions with the bacterial pathogen *Pseudomonas syringae*. *Plant Cell* 15 317–330. 12566575 10.1105/tpc.007591 PMC141204

[B59] Thimm O. Bläsing O. Gibon Y. Nagel A. Meyer S. Krüger P. (2004). mapman: a user-driven tool to display genomics data sets onto diagrams of metabolic pathways and other biological processes. *Plant J.* 37 914–939. 14996223 10.1111/j.1365-313x.2004.02016.x

[B60] Thomma B. P. H. J. Nürnberger T. Joosten M. H. A. J. (2011). Of PAMPs and effectors: the blurred PTI-ETI dichotomy. *Plant Cell* 23 4–15. 10.1105/tpc.110.082602 21278123 PMC3051239

[B61] Torres M. A. Jones J. D. Dangl J. L. (2006). Reactive oxygen species signaling in response to pathogens. *Plant Physiol.* 141 373–378. 16760490 10.1104/pp.106.079467 PMC1475467

[B62] Tsuda K. Katagiri F. (2010). Comparing signaling mechanisms engaged in pattern-triggered and effector-triggered immunity. *Curr. Opin. Plant Biol.* 13 459–465. 10.1016/j.pbi.2010.04.006 20471306

[B63] Van de Wouw A. P. Lowe R. G. Elliott C. E. Dubois D. J. Howlett B. J. (2014). An avirulence gene, *AvrLmJ1*, from the blackleg fungus, *Leptosphaeria maculans*, confers avirulence to *Brassica juncea* cultivars. *Mol. Plant Pathol.* 15 523–530. 10.1111/mpp.12105 24279453 PMC6638781

[B64] van Verk M. C. Bol J. F. Linthorst H. J. (2011). WRKY transcription factors involved in activation of SA biosynthesis genes. *BMC Plant Biol.* 11:89. 10.1186/1471-2229-11-89 21595875 PMC3120740

[B65] Wang X. Wang H. Wang J. Sun R. Wu J. Liu S. (2011). The genome of the mesopolyploid crop species *Brassica rapa*. *Nat. Genet.* 43:1035. 10.1038/ng.919 21873998

[B66] West J. S. Kharbanda P. Barbetti M. Fitt B. D. (2001). Epidemiology and management of *Leptosphaeria maculans* (phoma stem canker) on oilseed rape in Australia, Canada and Europe. *Plant Pathol.* 50 10–27.

[B67] Yang B. Jiang Y. Rahman M. H. Deyholos M. K. Kav N. N. (2009). Identification and expression analysis of WRKY transcription factor genes in canola (*Brassica napus* L.) in response to fungal pathogens and hormone treatments. *BMC Plant Biol.* 9:68. 10.1186/1471-2229-9-68 19493335 PMC2698848

[B68] Yasuda M. Ishikawa A. Jikumaru Y. Seki M. Umezawa T. Asami T. (2008). Antagonistic interaction between systemic acquired resistance and the abscisic acid–mediated abiotic stress response in *Arabidopsis*. *Plant Cell* 20 1678–1692. 10.1105/tpc.107.054296 18586869 PMC2483369

[B69] Yoshimoto K. Jikumaru Y. Kamiya Y. Kusano M. Consonni C. Panstruga R. (2009). Autophagy negatively regulates cell death by controlling NPR1-dependent salicylic acid signaling during senescence and the innate immune response in *Arabidopsis*. *Plant Cell* 21 2914–2927. 10.1105/tpc.109.068635 19773385 PMC2768913

[B70] Yu F. Lydiate D. J. Rimmer S. R. (2008). Identification and mapping of a third blackleg resistance locus in *Brassica napus* derived from *B. rapa* subsp. *sylvestris*. *Genome* 51 64–72. 18356940 10.1139/g07-103

[B71] Zhang C. Chen H. Cai T. Deng Y. Zhuang R. Zhang N. (2017). Overexpression of a novel peanut NBS-LRR gene *AhRRS5* enhances disease resistance to *Ralstonia solanacearum* in tobacco. *Plant Biotechnol. J.* 15 39–55. 10.1111/pbi.12589 27311738 PMC5253469

[B72] Zhang L. Kars I. Essenstam B. Liebrand T. W. Wagemakers L. Elberse J. (2014). Fungal endopolygalacturonases are recognized as microbe-associated molecular patterns by the *Arabidopsis* receptor-like protein RESPONSIVENESS TO BOTRYTIS POLYGALACTURONASES1. *Plant Physiol.* 164 352–364. 10.1104/pp.113.230698 24259685 PMC3875813

