# Supplementary material for: Transcriptional Insight Into Brassica napus Resistance Genes LepR3 and Rlm2-Mediated Defense Response Against the Leptosphaeria maculans Infection
Source: Front Plant Sci. 2019 Jul 2;10:823. doi: 10.3389/fpls.2019.00823 (PMC6615431; doi:10.3389/fpls.2019.00823)
Supplement: Supplementary file 1 [file Table_1.pdf]

Table S1. Sequence analysis of cloned *six Avr* genes in four *Leptosphaeria maculans* isolates.

	L. maculans isolates			
	03-42-06	87-41	03-15-03	PG4-1-M
AvrLm1[a]				
Genotype	A	A	a	a
PCR	+	+	-	-
AvrLm2[b]				
Genotype	a	A	A	a
PCR	+	+	+	+
Nucleotides for 133rd AA	CAT	GGT	GGT	AAT
Nucleotides for 146th AA	CAA	GAA	GAA	CAA
AvrLm4-7[c]				
Genotype	A	A	a	a
PCR	+	+	-	-
Nucleotides for 80th AA	ATA	ATA		
Nucleotides for 120th AA	GGC	GGC		
AvrLm6[d]				
Genotype	A	A	A	A
PCR	+	+	+	+
AvrLm11[e]				
Genotype	A	A	A	A
PCR	+	+	+	+
AvrLm5[f]				
Genotype	A	A	A	A
PCR	+	+	+	+
Nucleotides for 29th AA	CGA	CGA	CGA	CGA
Nucleotides for 38th AA	CGG	CGG	CGG	CGG
Nucleotides for 55th AA	AGA	AGA	AGA	AAA

a, d and e. Virulent allele of *AvrLm1, AvrLm6* or *AvrLm11* was caused by deletion of the gene; PCR was used to detect the presence/absence of *Avr* genes in four isolates.
b. Polymorphic sites (133rd and 146th amino acids) determining the avirulence/virulence toward *Rlm2* in *AvrLm2*. The nucleotides GGT coding Gly in 133rd and GAA coding Glu in 146th render the *Avrlm2* complementary and avirulent to *Rlm2* (Ghanbarnia *et al*., 2015).
c. Polymorphic sites (120th amino acid) determining the avirulence/virulence toward *Rlm4* in *AvrLm4-7*. The nucleotides GGC coding Gly for the 120th render the *AvrLm4-7* complementary and avirulent to *Rlm4* (Blondeau *et al.*, 2015).
f. Polymorphic sites (29th amino acid) in *AvrLm5* determining the avirulence/virulence toward *B. napus* cultivars . The nucleotide changes from CGA coding Arg for the 29th amino acid to TGA coding the premature stop code result in the virulence of *AvrLm5* toward *B. napus* cultivars (Van de Wouw *et al*., 2014). The polymorphic sites for 38th and 55th amino acids were also detected.
For genotype indication, 'A' means the presence of the functional *Avr* gene while 'a' means presence of the non-functional *Avr* gene or the absence of the *Avr* gene. For PCR screening, '+' means the detection of the *Avr* gene by PCR, '-' means the absence of the *Avr* gene in the PCR screening.
