# Supplementary material for: Transcriptional Insight Into Brassica napus Resistance Genes LepR3 and Rlm2-Mediated Defense Response Against the Leptosphaeria maculans Infection
Source: Front Plant Sci. 2019 Jul 2;10:823. doi: 10.3389/fpls.2019.00823 (PMC6615431; doi:10.3389/fpls.2019.00823)
Supplement: Supplementary file 5 [file Table_5.docx]

**Table S5. List of primers used in this study**

| Primer name | Sequence (5' -3') | Application |
| --- | --- | --- |
| AvrLm1NF | ATAGTCTGTGCGACCTA | Genotype detection |
| AvrLm1NR | TAGTTTCAGGAGGGATA | Genotype detection |
| AvrLm2NF | GACCCGGACGAATAACG | Genotype detection |
| AvrLm2NR | CCTACGCTTAACGACTCTA | Genotype detection |
| AvrLm47NF | GAACCCTGCTAGATAGG | Genotype detection |
| AvrLm47NR | GAAGTTAGGCTGATGGA | Genotype detection |
| AvrLm6NF | TAAACTTGATCCCACAT | Genotype detection |
| AvrLm6NR | CAGTTTTGAACCGTAGT | Genotype detection |
| AvrLm11NF | TACCTATTTACCCTATTTGC | Genotype detection |
| AvrLm11NR | TAAGTGTCCTCTGTGCC | Genotype detection |
| AvrLm5NF | TCCTTCGCTTAGTGATT | Genotype detection |
| AvrLm5NR | TGGCGATAGTACAGAAG | Genotype detection |
| Le3_Rl2_F | GCAGTAGAGGAACCCATCTGAGGA | Genotype detection |
| Le3_Rl2_R | CGAGTGTGTTCTTCAA | Genotype detection |
| 35S_Le3_F | GGAGTTAAUAAATTGTCAAGTCAACCATG | Genotype detection |
| 35S_Le3_R | GGTCTTAAUTTAACGACGTGTGGTGCTTA | Genotype detection |
| BnaC01g36910DF | GTCGAGAAACATATC | Real time PCR |
| BnaC01g36910DR | CGTCTTCGCCTCCCC | Real time PCR |
| BnaC05g39200DF | GCGAAAGTCGATATCA | Real time PCR |
| BnaC05g39200DR | AGGATGTGCCCATCA | Real time PCR |
| BnaA01g29390DF | GTCGAGAAGCATTTA | Real time PCR |
| BnaA01g29390DR | TCGTCTTCACCTCCCC | Real time PCR |
| BnaA05g25030DF | GCGAAAGTCGATCTCG | Real time PCR |
| BnaA05g25030DR | AGGATGTGCCCATCG | Real time PCR |
| BnaC01g25570DF | AACTGGAGCCGGTTCA | Real time PCR |
| BnaC01g25570DR | CTCTACGACCCAGTTG | Real time PCR |
| BnaA01g20340DF | ACAGGACATTCTTCAGGT | Real time PCR |
| BnaA01g20340DR | AGCGTGCTTGAAGACACT | Real time PCR |
| BnaA04g05420DF | AGCACTACGCAGGAT | Real time PCR |
| BnaA04g05420DR | CTCAAACCCAACAATT | Real time PCR |
| BnaA04g05430DF | GCCTTCTCCACATCA | Real time PCR |
| BnaA04g05430DR | AAATCCTGCGACTGA | Real time PCR |
| BnaC02g09180DF | TTGCTTTTGCTCTCGAT | Real time PCR |
| BnaC02g09180DR | CTTGCTCCAACAGGCT | Real time PCR |
| BnaA10g24230DF | TGTGATCGTTTCTGCGT | Real time PCR |
| BnaA10g24230DR | GGACTGAAGAGCTTGTTC | Real time PCR |
| BnaC09g48850DF | TCGTCTGCTACTTGTA | Real time PCR |
| BnaC09g48850DR | GATGATGTCCCAAACAGT | Real time PCR |
| BnaC04g56610DF | CGTCGAAGAATTGGGG | Real time PCR |
| BnaC04g56610DR | CTCTTGCCGAGTTTAA | Real time PCR |
| BnaC02g00690DF | GGATATCAGGAAGGATCTGTATGGAAA | Real time PCR |
| BnaC02g00690DR | CGAGCGCCGTGATCTCTTT | Real time PCR |
| Rlm2F | GCCTTGTTTTGACCGG | Real time PCR for *Rlm2* |
| Rlm2R | ATATGCAGTTGTCACTT | Real time PCR for *Rlm2* |
| Lepr3F | GCCTTGTTCCGGCTGG | Real time PCR for *LepR3* |
| Lepr3R | AGTGGCATCACATGC | Real time PCR for *LepR3* |
| blmr1-F | GCCTTGTTCTGACGAC | Real time PCR for *blmr1* |
| blmr1-R-LepR3 | AGTGGCATCACATCT | Real time PCR for *blmr1* |
| blmr1-R-Rlm2 | GGATGCAGTTGCCACCA | Real time PCR for *blmr1* |
