# Supplementary material for: Transcriptional Insight Into Brassica napus Resistance Genes LepR3 and Rlm2-Mediated Defense Response Against the Leptosphaeria maculans Infection
Source: Front Plant Sci. 2019 Jul 2;10:823. doi: 10.3389/fpls.2019.00823 (PMC6615431; doi:10.3389/fpls.2019.00823)
Supplement: Supplementary file 6 [file Data_Sheet_1.PDF]

```
                          (Signal peptide)
blmr1              1 MKGSVKSFSLIPISFCFLFLFRDEFAVPARHLC 33
Rlm2               1 MKGSVKSFSLIPISFCFLFLFRDEFAVPARNLC 33
LepR3              1 MKGSVKSFSLIPISFCFLFLFRDEFAVPARHLC 33

                     (Leucine rich repeat N-terminal domain)
blmr1             34 HPQQREAILELKNEFHIQKPCSDDRTVSWVNNSDCCSWDGIRCD ATFG 81
Rlm2              34 HPQQREAILELKNEFQIQKPCFD-RTVSWVNNSDCCSWDGIRCD ATFG 80
LepR3             34 HPQQREAILEFKNEFQIQKPCSG-WTVSWVNNSDCCSWDGIACD ATFG 80


blmr1             82 DVIELNLGGNCIHGELNSKNTILKLQSLP FLATLDLSDNYFSGNIPSSLGNLS 134
Rlm2              82 DVIELNLSDNCIYGQLNSKNTILKLQSLP FLATLDLSDNQLSGNVPSSLGNLS 133
LepR3             81 DVIELNLGGNCIHGELNSKNTILKLQSLP FLETLNLAGNYFSGNIPSSLGNLS 133


blmr1            135 KLTTLDLSDNDFNGEIPSSLGNLS      NLTTLDLSYNAFNGEIPSSLGNLS 182
Rlm2             134 KLTTLDLSENDFNGEIPSSLGNLY      NLTILNLSQNKLIGKIPPSLGNLS 181
LepR3            134 KLTTLDLSDNAFNGEIPSSLGKLY      NLTILNLSHNKLIGKIPSSFGRLK 181


blmr1            183 NLTILKLSQNKLIGKIPPSLGNLS      YLTHLTLCANNLVGEIPYSLANLSH 231
Rlm2                 ------------------------      YLTHLTLCANNLVGEIPYSLANLSH 206
LepR3                ------------------------      -------------------------


blmr1            232 HLTFLNICENSFSGEIPSFLGNFS      LLTLLDLSANNFVGEIPSSFGRLK 279
Rlm2             207 HLTFLSICENSFSGEIPSFLGNFS      LLTLLDLSANNFVGEIPSSFGRLK 254
LepR3                ------------------------      ------------------------


blmr1            280 HLTILSAGENKLTGNFPVT-LLNLT     KLLDLSLGYNQFTGMLPPNVSLLS 327
Rlm2             255 HLTILSAGENKLTGNFPVT-LLNLT     KLLDLSLGYNQFTGMLPPNVSLLS 302
LepR3            182 HLTGLYAADNELSGNFPVTTLLNLT     KLLSLSLYDNQFTGMLPPNISSLS 230


blmr1            328 NLEAFSIGGNALTGTLPSSLFSIP      SLTYVSLENNQLNGTLDFGNVSSSS 376
Rlm2             303 NLEAFSIGGNALTGTLPSSLFSIP      SLTYVSLENNQLNGTLDFGNVSSSS 351
LepR3            231 NLVAFYIRGNALTGTLPSSLFSIP      SLLYVTLEGNQLNGTLDFGNVSSSS 279


blmr1            377 KLMQLRLGNNNFLGSIPRAISKLV      NLDTLDLSHLNTQGSSVDLSILWNLK 426
Rlm2             352 KLMQLRLGNNNFLGSIPRAISKLV      NLDTLDLSHLNTQGSSVDLSILWNLK 401
LepR3            280 KLMQLRLGNNNFLGSIPRAISKLV      NLATLDLSHLNTQGLALDLSILWNLK 329


blmr1            427 SLVELDISDLNTTTAIDLNDILSRFK    WLDTLNLTGNHVTYEKRISVSDPP 476
Rlm2             402 SLVELDISDLNTTTAIDLNDILSRFK    WLDTLNLTGNHVTYEKRISVSDPP 451
LepR3            330 SLEELDISDLNTTTAIDLNAILSRYK    WLDKLNLTGNHVTYEKRSSVSDPP 379


blmr1            477 LLRDLYLSGCRFTTEFPGFIRTQH      NMEALDISNNKIKGQVPGWLWELS 524
Rlm2             452 LLRDLYLSGCRFTTEFPGFIRTQH      NMEALDISNNKIKGQVPGWLWELS 499
LepR3            380 LLSELYLSGCRFTTGFPELLRTQH      NMRTLDISNNKIKGQVPGWLWELS 427


blmr1            525 TLYYLNLSNNTFTSFESPNKLRQPS     SLYYFSGANNNFTGGIPSFICELH 573
Rlm2             500 TLYYLNLSNNTFTSFESPNKLRQPS     SLYYFSGANNNFTGGIPSFICELH 548
LepR3            428 TLEYLNISNNTFTSFENPKKLRQPS     SLEYLFGANNNFTGRIPSFICELR 476


blmr1            574 SLIILDLSSNRFNGSLPRCVGKFSS     VLEALNLRQNRLSGRLPKKIISR 621
Rlm2             549 SLIILDLSSNRFNGSLPRCVGKFSS     VLEALNLRQNRLSGRLPKKIISR 596
LepR3            477 SLTVLDLSSNKFNGSLPRCIGKFSS     VLEALNLRQNRLSGRLP-KIIFR 523


blmr1            622 GLKSLDIGHNKLVGKLPRSLIANS      SLEVLNVESNRFNDTFPSWLSSLP 669
Rlm2             597 GLKSLDIGHNKLVGKLPRSLIANS      SLEVLNVESNRFNDTFPSWLSSLP 644
LepR3            524 SLTSFDIGHNKLVGKLPRSLIANS      SLEVLNVESNRFNDTFPSWLSSLP 571
```

```
blmr1            670 ELQVLVLRSNAFHGPIHQTRFY        KLRIIDISHNRFNGTLPLDFFVNWT 716
Rlm2             645 ELQVLVLRSNAFHGPIHQTRFY        KLRIIDISHNRFNGTLPLDFFVNWT 691
LepR3            572 ELQVLVLRSNAFHGPVHQTRFS        KLRIIDISHNRFSGMLPSNFFLNWT 618

blmr1            717 SMHFIGKNGVQSNGNYMGTRRYYFDSMVLMNKGIEMELVRILYIYTALDFSENEFEGVIPSSIGLLK 783
Rlm2             692 SMHFIGKNGVQSNGNYMGTRRYYFDSMVLMNKGIEMELVRILYIYTALDFSENEFEGVIPSSIGLLK 758
LepR3            619 AMHSIGKDGDQSNGNYMGT-YYYFDSMVLMNKGVEMELVRILTIYTALDFSENEFEGVIPSSIGLLK 684

blmr1            784 ELHVLNLSGNAFTGRIPSSMGNLS      SLESLDLSRNKLTGEIPQELGNLS 831
Rlm2             759 ELHVLNLSGNAFTGRIPSSMGNLS      SLESLDLSRNKLTGAIPQELGNLS 758
LepR3            685 ELHVLNLSGNAFTGRIPSSMGNLS      SLESLDLSRNKLTGAIPQELGNLS 732

blmr1            832 YLAYMNFSHNQLVGLVPGGTQFR 854
Rlm2             759 YLAYMNFSHNQLAGLVPGGTQFR 829
LepR3            733 YLAYMNFSHNQLVGLVPGGTQFR 755

blmr1            855 TQPCSSFKDNPGLFGPSLNQACVD-IHGKTSQPSEMSKEEEEDGQEEVIS 903
Rlm2             830 TQPCSSFKDNPGLYGPSLEEVCVD-IHGKTSQPSELSKEEEEDGQEEVIS 878
LepR3            756 TQPCSSFKDNPGLFGPSLEEVCVDHIHGKTSQPSEMSKEEE-DGQEEVIS 804

                      (Transmembrane domain)
blmr1            904 WIAAAIGFIPGIAFGFTMEYIMVSYKPEWFINLFGRTKRRRISTTRR 950
Rlm2             879 WIAAAISFIPGIVFGFTMGHIVVSYKPQWFINPFGRTKRRRISTTRR 925
LepR3            805 WIAAAIGFIPGIVFGFTMGYIMVSYKPEWFINLFGRTKRRRISTTRR 851
```

Figure S1. Alignment and domain analysis of 'Westar' blmr1, 'Glacier' Rlm2 and 'Surpass 400' LepR3. The N-terminal signal peptide, the N-terminal leucine-rich repeat domain, and the C-terminal transmembrane domain are highlighted in gray. The leucine rich repeat motifs (LRRs) were identified using the LRRfinder in (http://www.lrrfinder.com/result.php) according to default parameters and contained in the red box, and the conservative hydrophobic amino acids were presented in bold, green front.
