# Supplementary material for: Transcriptional Insight Into Brassica napus Resistance Genes LepR3 and Rlm2-Mediated Defense Response Against the Leptosphaeria maculans Infection
Source: Front Plant Sci. 2019 Jul 2;10:823. doi: 10.3389/fpls.2019.00823 (PMC6615431; doi:10.3389/fpls.2019.00823)
Supplement: Supplementary file 7 [file Data_Sheet_2.PDF]

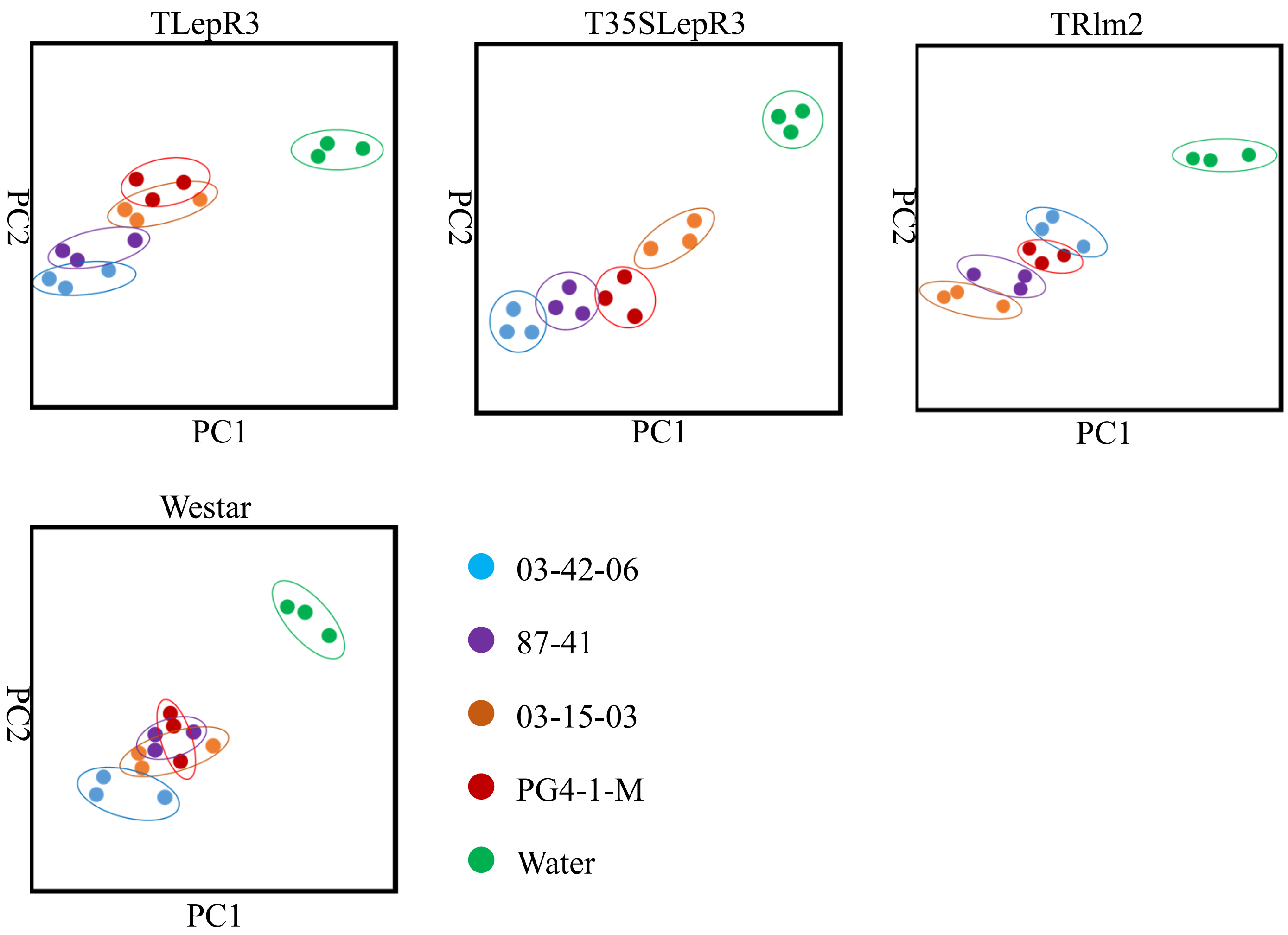

Figure S2. Principal component analysis of RNA-seq dataset collected on four different *B. napus* lines. Principal component analysis of RNA-seq dataset collected on four different *B. napus* lines including TLepR3, T35SLepR3, TRlm2 and 'Westar'. The analysis was performed on data collected on the same line inoculated with four *L. maculans* isolates 03-42-06, 03-15-03, 87-41 and PG4-1-M, and the water control was also put in the same analysis.
