# Supplementary material for: Transcriptional Insight Into Brassica napus Resistance Genes LepR3 and Rlm2-Mediated Defense Response Against the Leptosphaeria maculans Infection
Source: Front Plant Sci. 2019 Jul 2;10:823. doi: 10.3389/fpls.2019.00823 (PMC6615431; doi:10.3389/fpls.2019.00823)
Supplement: Supplementary file 8 [file Data_Sheet_3.PDF]

A

B. napus lines	*L. maculans* isolates	
Westar	03-42-06	'Basal' (0.05% DEG rate)
Westar	03-15-03	
TRlm2	03-42-06	'Comp' (0.3% DEG rate)
T35SLepR3	03-15-03	
TRlm2	03-15-03	'Incomp' (0.3% DEG rate)
T35SLepR3	03-42-06	

B

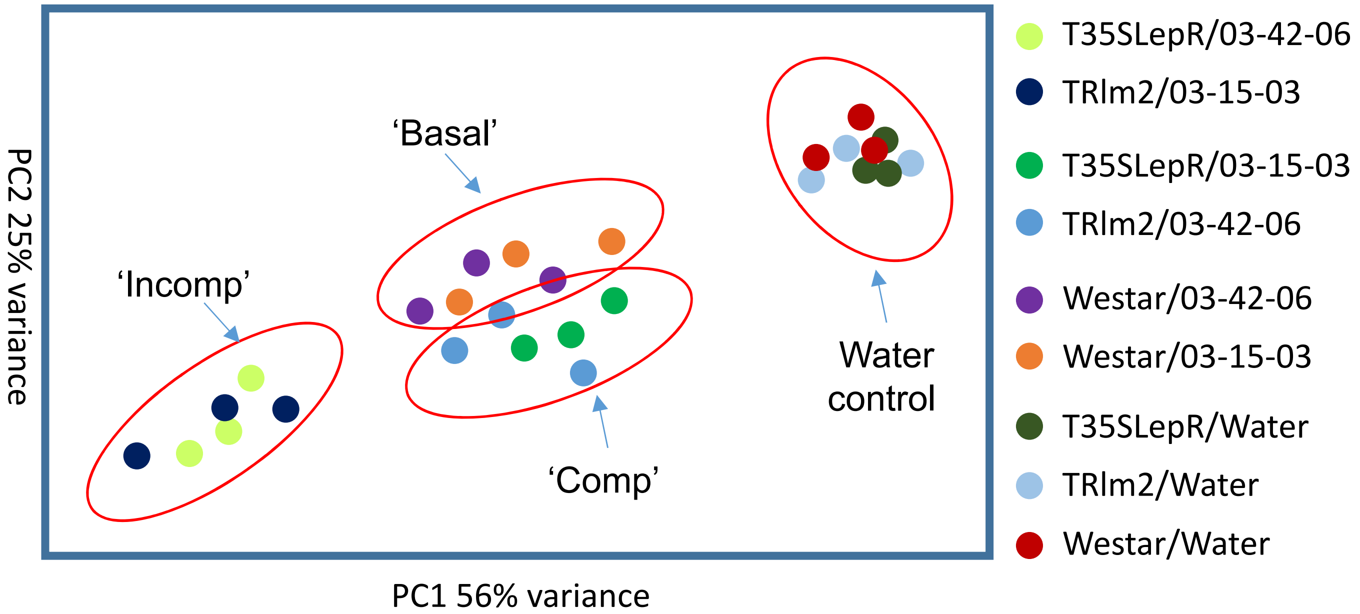

Figure S3. Classification and cluster of inoculation of 03-42-06 (*AvrLm1 & avrLm2*) and 03-15-03 (*avrLm1 & AvrLm2*) on 'Westar', T35SLepR3 and TRlm2. (A) According to presumed plant defense response levels from the compatible interaction on 'Westar' to the compatible interaction on T35SLepR3 and TRlm2 and to the incompatible interaction on T35SLepR3 and TRlm2, inoculation of 03-42-06 (*AvrLm1 & avrLm2*) and 03-15-03 (*avrLm1 & AvrLm2*) on 'Westar', T35SLepR3 and TRlm2 were classified into three groups, namely 'Basal', 'Comp' and 'Incomp'. Thanks to the low differentially expressed genes (DEG) rates between samples among the same group, RNA-seq data collected from the same group were pooled together for further analysis. In this arrangement, we are able to dissect the defense-mediated by *LepR3* and *Rlm2* into three levels, including basal defense in the susceptible 'Westar' background, defense response triggered by *LepR3* and *Rlm2* recognizing other fungal components rather than the cognate *Avr* genes and resistance response triggered by the *LepR3-AvrLm1* and *Rlm2-AvrLm2* recognition specificities. (B) Principal component analysis (PCA) of RNA-seq data among 'Basal', 'Comp', 'Incomp' and water inoculation on 'Westar', 'T35SLepR3' and 'TRlm2'. The PCA analysis revealed the RNA-seq sample among the same group are highly cluttered.
